# Development of a Highly Protective Combination Monoclonal Antibody Therapy against Chikungunya Virus

**DOI:** 10.1371/journal.ppat.1003312

**Published:** 2013-04-18

**Authors:** Pankaj Pal, Kimberly A. Dowd, James D. Brien, Melissa A. Edeling, Sergey Gorlatov, Syd Johnson, Iris Lee, Wataru Akahata, Gary J. Nabel, Mareike K. S. Richter, Jolanda M. Smit, Daved H. Fremont, Theodore C. Pierson, Mark T. Heise, Michael S. Diamond

**Affiliations:** 1 Department of Molecular Microbiology, Washington University School of Medicine, St. Louis, Missouri, United States of America; 2 Viral Pathogenesis Section, Laboratory of Viral Diseases, National Institute of Allergy and Infectious Diseases, National Institutes of Health, Bethesda, Maryland, United States of America; 3 Department of Medicine, Washington University School of Medicine, St. Louis, Missouri, United States of America; 4 Department of Biochemistry and Molecular Biophysics, Washington University School of Medicine, St. Louis, Missouri, United States of America; 5 MacroGenics, Rockville, Maryland, United States of America; 6 Vaccine Research Center, National Institute of Allergy and Infectious Diseases, National Institutes of Health, Bethesda, Maryland, United States of America; 7 Department of Medical Microbiology, Molecular Virology Section (HPC EB88), University Medical Center Groningen and University of Groningen, Groningen, Netherlands; 8 Department of Pathology & Immunology, Washington University School of Medicine, St. Louis, Missouri, United States of America; 9 Department of Genetics, University of North Carolina at Chapel Hill, Chapel Hill, North Carolina, United States of America; Institut Pasteur, France

## Abstract

Chikungunya virus (CHIKV) is a mosquito-transmitted alphavirus that causes global epidemics of a debilitating polyarthritis in humans. As there is a pressing need for the development of therapeutic agents, we screened 230 new mouse anti-CHIKV monoclonal antibodies (MAbs) for their ability to inhibit infection of all three CHIKV genotypes. Four of 36 neutralizing MAbs (CHK-102, CHK-152, CHK-166, and CHK-263) provided complete protection against lethality as prophylaxis in highly susceptible immunocompromised mice lacking the type I IFN receptor (*Ifnar^−/−^*) and mapped to distinct epitopes on the E1 and E2 structural proteins. CHK-152, the most protective MAb, was humanized, shown to block viral fusion, and require Fc effector function for optimal activity *in vivo*. In post-exposure therapeutic trials, administration of a single dose of a combination of two neutralizing MAbs (CHK-102+CHK-152 or CHK-166+CHK-152) limited the development of resistance and protected immunocompromised mice against disease when given 24 to 36 hours before CHIKV-induced death. Selected pairs of highly neutralizing MAbs may be a promising treatment option for CHIKV in humans.

## Author Summary

Chikungunya virus (CHIKV) is a mosquito-transmitted alphavirus that causes outbreaks of polyarthritis in humans, and is currently a threat to spread to the United States due to the presence of its mosquito vector, *Aedes albopictus*. At present, there is no licensed human vaccine or therapeutic available to protect against CHIKV infection. The primary goal of this study was to develop an antibody-based therapeutic agent against CHIKV. To do this, we developed a panel of 230 new mouse anti-CHIKV MAbs and tested them for their ability to neutralize infection of different CHIKV strains in cell culture. We identified 36 MAbs with broad neutralizing activity, and then tested several of these for their ability to protect immunocompromised *Ifnar* ^−/−^ mice against lethal CHIKV infection. In post-exposure therapeutic trials, administration of a single dose of a combination of two neutralizing MAbs limited the development of resistance and protected *Ifnar* ^−/−^ mice against disease even when given just 24 to 36 hours before CHIKV-induced death. Analogous protection against CHIKV-induced arthritis was seen in a disease model in wild type mice. Our data suggest that pairs of highly neutralizing MAbs may be a therapeutic option against CHIKV infection.

## Introduction

Chikungunya virus (CHIKV) infection causes a severe febrile illness in humans that is characterized by a debilitating polyarthritis, which can persist for months and cause significant morbidity [1], [2]. There are three genotypes of CHIKV: Asian, East/Central/South African (ECSA), and West African [3]–[5], with 95.2 to 99.8% amino acid identity [4]. The CHIKV strains from the recent epidemics belong to the ECSA genotype and have affected millions in Africa and the Indian subcontinent [3], [6]. Imported cases in the United States and outbreaks in Europe highlight the threat of CHIKV to developed countries [7]. Currently, there are no approved vaccines or therapeutics for CHIKV [8].

CHIKV is an enveloped alphavirus of the *Togaviridae* family that enters cells via receptor-mediated internalization and a low pH-triggered type II membrane fusion event in early endosomes. The mature virion is comprised of three structural proteins: a nucleocapsid protein and two glycoproteins, E1 and E2, where E2 functions in attachment to cells and E1 participates in virus fusion. Each 700 Å CHIKV virion contains 240 copies of the envelope and capsid proteins, which are arranged in T = 4 quasi-icosahedral symmetry. E1-E2 heterodimers assemble into 80 trimeric spikes on the virus surface [9]. X-ray crystallographic structures of the precursor pE3-E2-E1, mature E2-E1, and E1 proteins [10]–[13] have elucidated the architecture of the glycoprotein shell. The E1 ectodomain consists of three domains. Domain I (DI) is located between DII and DIII, the latter of which adopts an immunoglobulin-like fold. The fusion peptide is located at the distal end of DII. E1 monomers lie at the base of the surface spikes and form a trimer around each of the icosahedral axes. E2 localizes to a long, thin leaf-like structure on the top of the spike. The mature E2 protein contains three domains with immunoglobulin-like folds: the N-terminal domain A, located at the center; domain B at the tip; and the C-terminal domain C, located proximal to the viral membrane.

Mouse models have been developed for CHIKV infection. Newborn outbred and inbred mice are vulnerable to severe CHIKV infection with viral replication observed in muscle, joint, and skin [14], [15]. Adult mice with defects in type I interferon signaling (*Ifnar* ^−/−^ mice) develop lethal disease, with muscle, joint, and skin appearing as the primary sites of infection [15]. CHIKV infection of juvenile C57BL/6 mice by a subcutaneous route results in metatarsal foot swelling with histological evidence of arthritis, tenosynovitis and myositis [16], [17].

Passive transfer of MAbs or immune sera can protect animals against infection of alphaviruses including Sindbis (SINV), Semliki Forest (SFV), and Venezuelan equine encephalitis (VEEV) viruses [18]–[25]. Immune γ-globulin from human donors in the convalescent phase of CHIKV infection exhibited neutralizing activity *in vitro* and had partial therapeutic efficacy in *Ifnar* ^−/−^ and neonatal wild type mice when administered up to 24 hours after infection [26]. Although mouse and human MAbs that neutralize CHIKV infection have been reported [27], [28], their post-exposure efficacy against lethal infection *in vivo* has not been clearly established [29].

Here, we investigated the molecular basis of antibody-mediated neutralization of CHIKV using a panel of 230 newly generated, cloned MAbs. CHK-152 protected mice against CHIKV-induced mortality and disease. The inclusion of a second MAb (CHK-166 or CHK-102) prevented the emergence of viral resistance and extended the treatment window in *Ifnar* ^−/−^ mice up to 24 to 36 hours prior to death of the animals. Our results suggest that combination therapy with selected neutralizing MAbs has potential for treatment of CHIKV infection in humans.

## Results

### Generation of MAbs

We generated a panel of neutralizing MAbs against CHIKV as a first step towards a possible therapy in humans. We infected adult C57BL/6 mice deficient for interferon regulatory factor 7 (*Irf7* ^−/−^) with 10^4^ PFU of the La Reunion 2006 OPY-1 strain of CHIKV (CHIKV-LR); these mice were boosted with CHIK virus-like particles [30], soluble recombinant CHIKV E2 protein, or live CHIKV-LR. We immunized *Irf7* ^−/−^ rather than wild type (WT) mice, as CHIKV replicated to higher titers, induced stronger neutralizing antibody responses, yet did not cause lethal infection in these innate immune-deficient animals ([31], and data not shown). We screened four independent myeloma cell-splenocyte fusions for binding of hybridoma supernatants to CHIKV-LR infected cells (**Fig. S1**) and cloned 230 CHIKV-specific MAbs for further analysis (**Table S1** in **Text S1**). Using a single endpoint neutralization assay, we identified 36 MAbs with inhibitory activity against infection of CHIKV-LR in BHK21-15 cells (data not shown).

### Neutralizing activity

**Figure 1:**
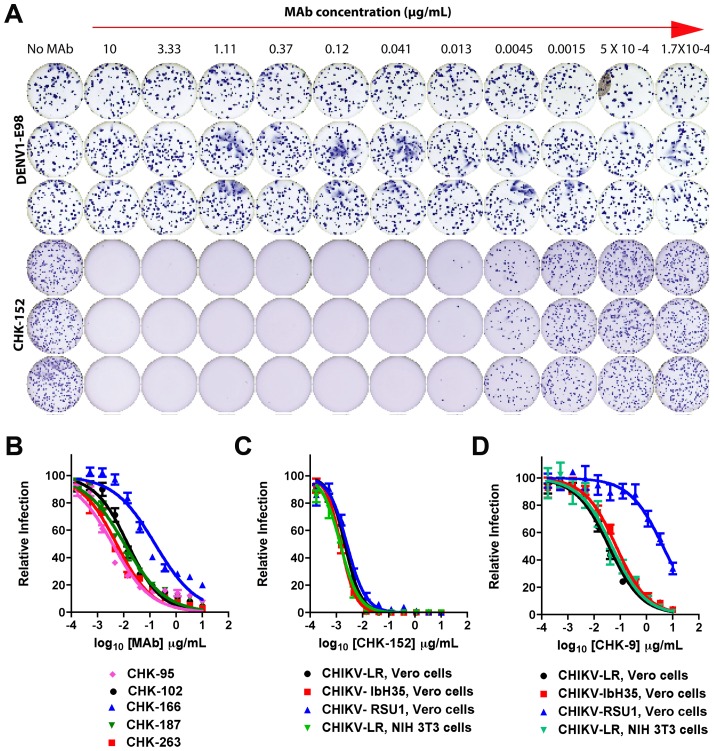
Profile of neutralizing MAbs against CHIKV. **A**. Examples of MAb neutralization as judged by a reduction in the number of FFU using the Biospot Macroanalyzer. Rows 2 to 12 going across represent decreasing (3-fold) concentrations of CHK-152 or the negative control DENV1-E98 MAb. Column 1 shows infection in the absence of MAb. **B**. Increasing concentrations of CHK-95, CHK-102, CHK-166, CHK-187, or CHK-263 were mixed with 100 to 150 FFU of CHIKV-LR for one hour at 37°C and Vero cells were infected. Neutralization was determined by FFU assay. **C–D**. CHK-152 (**C**) or CHK-9 (**D**) was mixed with CHIKV-LR (East, Central and South African genotype), CHIKV-RSUI (Asian genotype), or CHIKV IbH35 (West African genotype) for one hour at 37°C and Vero or NIH 3T3 cells were infected as indicated. Neutralization was determined by FFU assay. Data in this Figure is pooled from three independent experiments performed in duplicate or triplicate. All error bars represent the standard deviations.

To assess the inhibitory potential of our anti-CHIKV MAbs against the homologous CHIKV-LR and representative strains from the Asian and West African genotypes (RSU1 and IbH35 respectively), we performed focus reduction neutralization tests (FRNTs) on Vero cells. We determined the concentration of MAb that reduced the number of foci of infection by 50 or 90% (EC50 and EC90 values, **Fig. 1A and B**, **and** **Table 1**). CHK-152 was the most strongly neutralizing MAb we identified; 3 and 15 ng/ml of this MAb prevented 50 and 90% of CHIKV infection against all three CHIKV genotypes (**Fig. 1C**). Ten other MAbs inhibited CHIKV infection with EC50 values of <10 ng/ml against all three genotypes, and many others inhibited all three strains similarly, with a few exceptions. For example, CHK-9 failed to neutralize the Asian strain to the same extent as the West African or La Reunion (ECSA genotype) strains (**Fig. 1D**), whereas CHK-151 inhibited infection of the Asian strain better than the others (**Table 1**). Also, for reasons that are unclear, some neutralizing MAbs (e.g., CHK-143, CHK-264, and CHK-269) were incapable of inhibiting all viruses (EC90>10,000 ng/ml) in this assay, even at high MAb concentrations.

**Table 1:** Inhibitory activity of neutralizing anti-CHIKV MAbs.

MAb	CHIKV-LR EC50 ng/ml (CI)	CHIKV-LR EC90 ng/ml (CI)	CHIKV-LR EC50 ng/ml (CI)	CHIKV-RSU1 EC50 ng/ml (CI)	CHIKV-RSU1 EC90 ng/ml (CI)	CHIKV-IbH35 EC50 ng/ml (CI)	CHIKV IbH35 EC90 ng/ml (CI)
	Vero	Vero	3T3	Vero	Vero	Vero	Vero
**CHK-9**	36 (31–43)	882 (612–1271)	37 (27–51)	3712 (2636–5229)	**>10,000**	68 (54–87)	1478 (885–2469)
**CHK-11**	1356 (1049–1753)	**>10,000**	4680 (3691–5932)	215 (150–308)	**>10,000**	1141 (570–2284)	>10,000
**CHK-48**	16 (14–20)	430 (304–607)	32 (17–61)	***8*** (7–11)	220 (121–399)	***7*** (5–9)	359 (193–669)
**CHK-65**	**7** (6–9)	587 (330–1047)	25 (15–43)	***5*** (4–6)	265 (164–431)	***4*** (3–5)	147 (80–271)
**CHK-77**	48 (41–56)	576 (407–817)	91 (53–155)	17 (14–20)	368 (250–543)	27 (22–32)	444 (291–677)
**CHK-88**	***5*** (3–6)	422 (230–776)	12 (7–21)	***2*** (2–3)	190 (106–343)	***2*** (2–3)	211 (104–431)
**CHK-95**	***4*** (3–5)	156 (90–271)	***6*** (4–10)	***6*** (5–8)	411 (246–686)	***2*** (1–3)	176 (76–404)
**CHK-96**	40 (33–50)	8457 (5115–13982)	**>10,000**	95 (65–139)	**>10,000**	19 (14–26)	4486 (2126–9466)
**CHK-98**	155 (130–183)	5337 (3630–7846)	394 (220–706)	21 (16–27)	2022 (950–4304)	103 (80–131)	7923 (4494–13968)
**CHK-102**	14 (11–17)	351 (227–543)	30 (17–53)	***5*** (4–7)	1318 (532–3261)	***6*** (5–7)	104 (68–161)
**CHK-105**	19 (16–23)	1156 (773–1728)	94 (59–150)	11 (9–14)	927 (563–1528)	11 (9–13)	645 (433–960)
**CHK-112**	11 (9–13)	235 (162–339)	12 (8–19)	***8*** (6–11)	2116 (1072–4174)	***4*** (3–5)	115 (76–176)
**CHK-124**	***4*** (4–6)	101 (66–156)	***10*** (5–22)	***3*** (2–4)	228 (129–402)	***3*** (2–3)	96 (55–167)
**CHK-140**	***9*** (8–11)	219 (137–348)	56 (32–98)	***8*** (6–11)	721 (345–1508)	***5*** (4–6)	83 (55–125)
**CHK-142**	***9*** (7–11)	369 (238–572)	32 (21–48)	***7*** (5–10)	731 (321–1668)	***4*** (3–5)	108 (66–177)
**CHK-143**	34 (23–51)	**>10,000**	277 (76–1010)	***6*** (5–9)	3456 (1332–8965)	12 (7–23)	3094 (728–13143)
**CHK-151**	6883 (3467–13665)	**>10,000**	**>10,000**	261(154–440)	**>10,000**	5784 (2785–12015)	**>10,000**
**CHK-152**	***2*** (2–2)	***10*** (9–12)	***1*** (1–2)	***3*** (2–3)	15 (11–22)	***1*** (1–2)	***6*** (5–8)
**CHK-155**	***5*** (5–7)	110 (73–166)	***7*** (6–8)	***4*** (4–5)	93 (61–142)	***2*** (2–3)	52 (32–86)
**CHK-164**	3523 (2904–4274)	**>10,000**	4395 (3045–6342)	1637 (1182–2265)	**>10,000**	2366 (1582–3538)	**>10,000**
**CHK-165**	9725 (6275–15070)	**>10,000**	>10,000	1817 (111–2972)	**>10,000**	**>10,000**	**>10,000**
**CHK-166**	154 (116–205)	8604 (4459–16604)	202 (98–418)	40 (30–52)	2175 (1195–3959)	82 (59–114)	2576 (1234–5379)
**CHK-175**	***6*** (5–8)	423 (285–626)	***5*** (3–10)	***3*** (3–4)	343 (199–593)	***4*** (3–5)	662 (295–1489)
**CHK-176**	806 (616–1055)	**>10,000**	**>10,000**	**>10,000**	**>10,000**	**>10,000**	**>10,000**
**CHK-180**	140 (104–186)	7615 (3907–14843)	168 (74–384)	49 (38–63)	2588 (1465–4570)	79 (58–108)	1597 (802–3180)
**CHK-187**	11 (9–12)	524 (355–772)	17 (8–34)	***7*** (6–8)	140 (97–203)	***5*** (4–7)	186 (91–383)
**CHK-189**	4325 (3172–5897)	**>10,000**	3974 (2119–7451)	951 (589–1538)	**>10,000**	3809 (1856–7816)	**>10,000**
**CHK-262**	4934 (3938–6181)	**>10,000**	3740 (2488–5623)	4622 (3376–6328)	**>10,000**	3306 (2049–5336)	**>10,000**
**CHK-263**	***5*** (5–7)	136 (93–197)	***2*** (2–4)	***4*** (3–5)	48 (32–73)	***2*** (2–2)	51 (36–74)
**CHK-264**	18 (13–23)	**>10,000**	***6*** (4–10)	***9*** (7–14)	1019 (430–2419)	11 (7–16)	5962 (2099–16932)
**CHK-265**	***8*** (7–9)	207 (148–289)	***6*** (4–8)	***5*** (4–6)	143 (88–234)	**5** (4–6)	201 (103–389)
**CHK-266**	2187 (1642–2913)	**>10,000**	1500 (1093–2059)	**>10,000**	**>10,000**	1330 (814–2173)	**>10,000**
**CHK-267**	18 (14–23)	1063 (628–1799)	***3*** (3–5)	***9*** (7–11)	452 (262–780)	14 (6–34)	3694 (468–29151)
**CHK-268**	24 (19–30)	934 (567–1540)	***10*** (7–14)	22 (17–29)	1212 (641–2289)	***10*** (8–13)	468 (278–789)
**CHK-269**	136 (104–178)	**>10,000**	32 (18–57)	50 (33–76)	**>10,000**	47 (33–69)	3834 (1617–9090)
**CHK-270**	14 (10–21)	9302 (3777–22912)	N.D.	14 (10–18)	669 (340–1317)	***8*** (6–11)	748 (363–1537)

Neutralizing activity was determined by FRNT on Vero or NIH 3T3 cells with increasing concentrations of purified MAbs and 100 FFU of the indicated CHIKV strains corresponding to different genotypes (CHIKV-LR, East, Central, and South African; CHIKV-RSUI, Asian and, IbH35, West African). The data were derived from three independent experiments performed in duplicate or triplicate. The inhibitory concentrations of MAb that reduced infected foci by 50% (EC50) and 90% (EC90) were calculated by nonlinear regression analysis and are expressed as ng/ml of antibody. In parenthesis, immediately below the EC50 and EC90 values, are confidence intervals (CI). Bold indicates that the EC50 or EC90 value was greater than the highest concentration (10,000 ng/ml) of MAb used. Bold and italicized indicates an EC50 or EC90 value of less than or equal to 10 ng/ml, which reflects a highly neutralizing MAb for a given cell type or virus strain. N.D. indicates not determined. Of note, NIH 3T3 cells are less permissive than Vero cells (1 FFU on NIH 3T3 cells = 35 FFU on Vero cells).

We speculated that some MAbs might show cell type-dependent neutralization if they blocked attachment to cell type-specific factors. To test this hypothesis, we assessed MAb neutralization of CHIKV-LR infection in cells of another species, NIH 3T3 mouse fibroblasts (**Table 1**). For most MAbs, the EC50 values were comparable to those achieved with Vero cells. However, two MAbs (CHK-96 and CHK-176) showed a 12 to 250-fold reduction (*P*<0.05) in neutralizing activity on NIH 3T3 compared to Vero cells; although further study is warranted, these MAbs may block a step in the entry pathway that varies among different cell types.

### Prophylaxis studies

**Figure 2:**
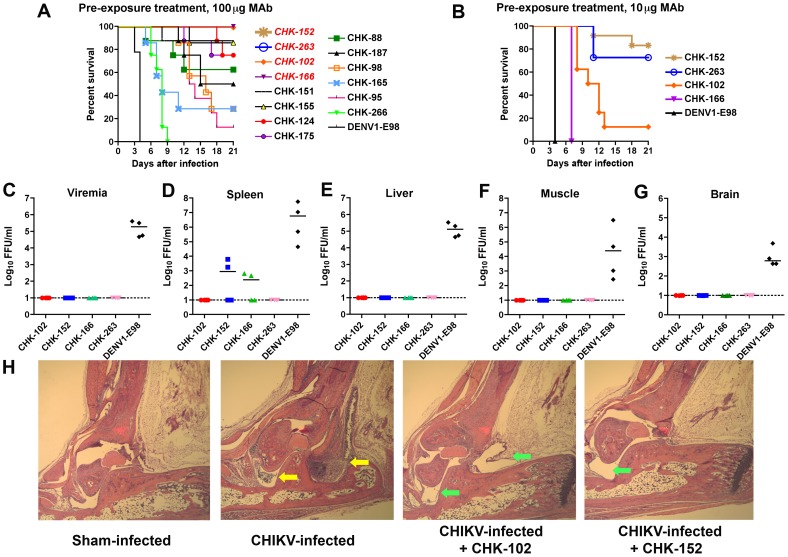
Efficacy of anti-CHIKV MAb prophylaxis. **A**. Six to eight week-old *Ifnar* ^−/−^ C57BL/6 mice were passively transferred 100 µg of the indicated MAbs via an i.p. injection one day before infection with 10 FFU of CHIKV-LR via a s.c. route. The percentage and number of surviving mice were as follows: DENV1-E98 (0%, 0 of 9), CHK-88 (62.5%; 5 of 8), CHK-95 (12.5%; 1 of 8), CHK-98 (28.6%; 2 of 7), CHK-102 (100%; 8 of 8), CHK-124 (75%; 6 of 8), CHK-151 (87.5%; 7 of 8), CHK-152 (100%; 8 of 8), CHK-155 (85.7%; 6 of 7), CHK-165 (28.6%; 2 of 7), CHK-166 (100%; 8 of 8), CHK-175 (75%; 6 of 8), CHK-187 (50%; 4 of 8), CHK-263 (100%; 8 of 8), or CHK-266 (0%; 0 of 8). MAbs italicized in red in the Figure provided 100% protection. **B**. *Ifnar* ^−/−^ mice were passively transferred 10 µg of MAb via an i.p. injection one day before infection with 10 FFU of CHIKV-LR via a s.c. route. The percentage and number of surviving mice were as follows: DENV1-E98 (0%; 0 of 7), CHK-102 (12.5%; 1 of 8), CHK-152 (83%; 10 of 12), CHK-166 (0%; 0 of 12), or CHK-263 (73%; 8 of 11). For (**A**) and (**B**) the survival curves were constructed from data of at least two independent experiments. All anti-CHK MAbs provided statistically significant protection in the percentage of surviving animals or mean survival time compared to the control DENV1-E98 MAb (*P*<0.05). **C–G**. Viral burden in MAb-treated *Ifnar* ^−/−^ mice. Animals were passively transferred 100 µg of the indicated MAbs (CHK-102, CHK-152, CHK-166, CHK-263, or isotype control DENV1-E98) via an i.p. injection one day before infection with 10 FFU of CHIKV-LR via a s.c. route. Two days later, viremia (**C**) and tissues (**D**, spleen; **E**, liver; **F**, muscle; and **G**, brain) were harvested and infectious virus was titrated by focus-forming assay. Results are pooled from two independent experiments (*n* = 4 mice per group). The dashed line indicates the limit of detection of the assay and the solid bar indicates the median values. All viral burden results with CHK-102, CHK-152, CHK-166, and CHK-263 were statistically different (*P*<0.02) from those obtained with DENV1-E98, as analyzed by the Mann-Whitney test. **H**. Four week-old female WT C57BL/6 mice were sham-treated or administered 100 µg of CHK-102 or CHK-152 via an i.p. route. 24 hours later, mice were infected with 100 PFU of CHIKV-SL 15649 and at day 10, virus-induced pathology in the foot and ankle joint was assessed. (*Outer left*) Sham-infected, (*middle left*) CHIKV infected and sham-treated, (*middle right*) CHIKV-infected and CHK-102 treated, and (*outer right*) CHIKV infected and CHK-152 treated. Shown are representative images after hematoxylin and eosin staining from at least 3 mice per group at 100× magnification. Yellow and green arrows indicate regions of inflammation or normal joints, respectively.

To evaluate whether neutralizing MAbs protect against CHIKV infection *in vivo*, we initially used a stringent test model: prevention of lethal infection in immunodeficient *Ifnar* ^−/−^ C57BL/6 mice. One hundred micrograms of 14 different MAbs with strong, modest, or poor neutralizing activity were administered to *Ifnar* ^−/−^ mice one day prior to CHIKV-LR infection. As seen previously [15], all *Ifnar* ^−/−^ mice died by day 4 after infection when treated with saline or a negative control MAb (**Fig. 2A**, and data not shown). Strongly neutralizing (e.g., CHK-102, CHK-152, and CHK-263) and one moderately inhibitory (CHK-166) MAb protected 100% of mice from lethal infection (*P*<0.0001). In comparison, and somewhat surprisingly, CHK-95, a potently neutralizing MAb of the same IgG2c isotype, protected only 12% of mice from death. The other MAbs tested conferred intermediate levels of protection (**Fig. 2A**). Thus, although several strongly neutralizing MAbs prevented against lethal CHIKV infection in *Ifnar* ^−/−^ mice, *in vitro* neutralization activity *per se* did not directly correlate with protection. To define the relative potency of the four MAbs that completely prevented lethal disease, we administered a lower (10 µg) dose. Whereas CHK-152 and CHK-263 still protected most mice from lethal infection, CHK-102 and CHK-166 protected to a lesser degree or only prolonged survival (**Fig. 2B**). Consistent with their ability to protect against lethal infection, passive transfer of CHK-102, CHK-152, CHK-166, and CHK-263 MAbs all markedly reduced viral loads in serum, spleen, liver, muscle, and brain at 48 hours after infection relative to a non-binding isotype control (DENV1-E98) MAb (**Fig. 2C–G**). The level of protection afforded by CHK-102, CHK-152, CHK-166, and CHK-263 MAbs, however, did not correlate directly with their binding strength to CHIKV surface glycoproteins (**Fig. S2**).

Although a stringent test of MAb protection, CHIKV-infected *Ifnar* ^−/−^ mice do not develop the arthritis observed in humans. To evaluate this, we utilized a WT C57BL/6 mouse model in which inoculation of CHIKV into the footpad results in localized swelling and induction of arthritis and fasciitis within the foot and ankle [16], [17], although infection does not cause lethality. Pretreatment of mice with either 100 µg of CHK-102 or CHK-152 completely protected against CHIKV-induced swelling, compared to control animals, which developed clinically apparent swelling (data not shown). While CHIKV infected control animals developed inflammatory arthritis in the ankle and foot, CHK-102 or CHK-152 MAb treated animals had normal appearing joint tissues (**Fig. 2H**).

### Mechanism of neutralization

**Figure 3:**
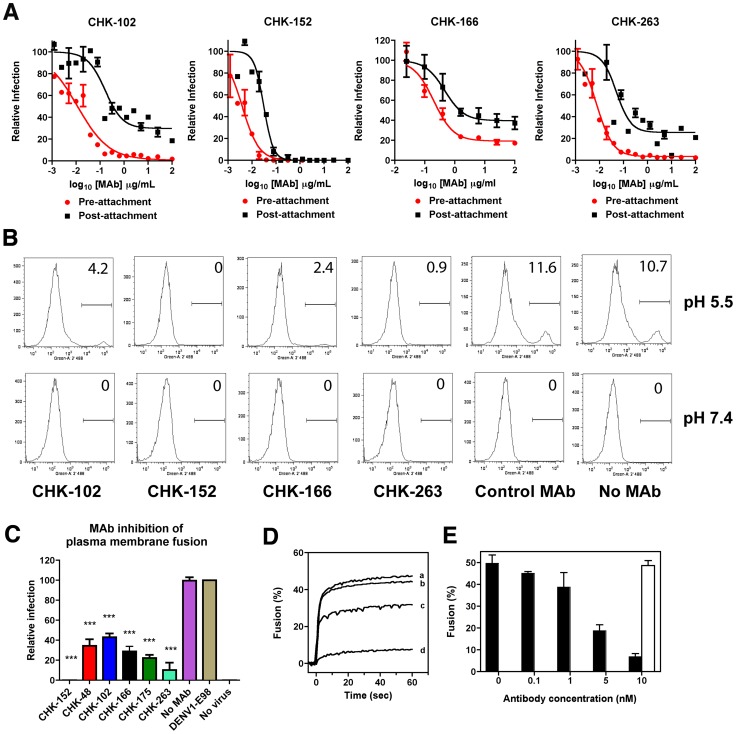
Mechanism of neutralization by CHIKV MAbs. **A**. Pre- and post-attachment inhibition assays. Vero cells were pre-chilled to 4°C and 100 FFU of CHIKV-LR was added to each well for one hour. After extensive washing at 4°C, the indicated MAbs were added for one hour at 4°C, and then the FRNT protocol was completed (*black lines*, *Post*). In comparison, a standard pre-incubation FRNT with all steps performed at 4°C is shown for reference. Virus and MAb are incubated together for one hour at 4°C, prior to addition to cells (*red lines, Pre*). Data shown are representative of three experiments performed in duplicate with error bars representing standard deviation. **B–C**. FFWO assay. CHIKV was incubated with Vero cells at 4°C to allow virus attachment. Free virus was removed after washing and 50 µg/ml of the indicated MAbs (including DENV1-E98, a negative control MAb) were added at 4°C. Viral fusion at the plasma membrane was induced after a brief exposure to a low pH buffer. After pH normalization, cells were cultured for 14 hours in the presence of NH_4_Cl to inhibit infection through the endosomal pathway. Cells were analyzed for infection by staining with an anti-E2 MAb. Representative histograms are shown (**B**) and the data was pooled from four independent experiments for statistical analysis (**C**). For simplicity of display, not all of the MAbs included in the summary graph are shown by flow cytometry analysis. Asterisks indicate values that are statistically different (*P*<0.05) from the control MAb. Error bars represent standard deviations. Note low pH-triggered viral fusion at the plasma membrane is an inefficient process with only 10 to 20% of cells becoming infected even when a high MOI was used. **D–E**. Viral membrane fusion with liposomes. Fusion of pyrene-labeled CHIKV was measured at pH 4.7 (37°C) using liposomes consisting of phosphatidylcholine, phosphatidylethanolamine, sphingomyelin, and cholesterol in a molar ratio of (1/1/1/1.5), as described in the Methods. (**D**) Curve a, no MAb; curve b, 0.1 nM CHK-152; curve c, 1 nM CHK-152; curve d, 10 nM CHK-152. (**E**) Extent of fusion (average value between 50 to 60 seconds post acidification) at increasing concentrations of MAb. Black bars, CHK-152; white bar, isotype control (MAb 0031, only included at 10 nM concentration). All fusion measurements were performed at least three independent times.

Antibody neutralization of enveloped viruses can occur by inhibiting attachment, internalization, and/or fusion [32], [33]. To determine how many of our most protective MAbs inhibited infection in cell culture, we performed pre- and post-attachment neutralization assays [34], [35]. Anti-CHK MAbs were incubated with CHIKV before or after virus binding to cells, and infection was measured. As expected, all MAbs efficiently neutralized infection when pre-mixed with virus (**Fig. 3A**). While CHK-102, CHK-152, CHK-166, and CHK-263 also inhibited CHIKV infection when added after virus adsorption to the cell surface, suggesting that at least part of their blocking activity was at a post-attachment step, differences in the extent of neutralization were noted in this context for several MAbs. CHK-152 completely neutralized all CHIKV virions without a resistant fraction when added post-attachment. When studies were repeated with eight other neutralizing MAbs that showed pre-exposure protection *in vivo*, no other MAb inhibited infection completely when added after virus adsorption to the cell. As expected, an isotype control MAb (DENV1-E98) and a non-neutralizing anti-CHK MAb (CHK-84) had no inhibitory effects in this assay (**Fig. S3**).

### Blockade of viral fusion

Since CHK-152 neutralized infection efficiently at a post-attachment step, we investigated whether it blocked fusion using a viral fusion from without (FFWO) assay [36]. CHIKV was adsorbed to Vero cell monolayers on ice and then treated with MAbs. Fusion at the plasma membrane was triggered after a brief exposure to low pH buffered medium at 37°C. Subsequently, cells were incubated in the presence of 20 mM NH_4_Cl to prevent CHIKV fusion via canonical endosomal pathways. As expected, at 14 hours after initial treatment, CHIKV infection was not observed when adsorbed virus was incubated at neutral pH (**Fig. 3B**). In comparison, in the absence of MAb or in the presence of a control MAb, a short exposure of cell surface-adsorbed virus to acidic pH resulted in infection and CHIKV-antigen positive cells. Notably, CHK-152 completely inhibited (*P*<0.0001) plasma membrane fusion and infection, whereas other anti-CHIKV neutralizing MAbs showed significant yet incomplete inhibition in this assay (**Fig. 3B and C**). These studies suggest that CHK-152 efficiently neutralizes infection by preventing the structural changes on the virion necessary for viral fusion with host cell membranes.

We utilized a model liposome fusion assay with pyrene-labeled virus [37], [38] to confirm these results. Pyrene-labeled CHIKV was pre-incubated with different concentrations of MAb, mixed with liposomes at 37°C, and fusion was triggered by addition of a low-pH buffer [37]. In the absence of MAb or in the presence of 10 nM (1.5 µg/ml) of a non-binding control MAb, fusion was complete within seconds of acidification. In contrast, pre-incubation of virus with increasing doses of CHK-152 inhibited fusion (**Fig. 3D and E**). Thus, CHK-152 can block low-pH-induced fusion of virus with liposomes.

### The effector functions of CHK-152 contribute to protection *in vivo*

**Figure 4:**
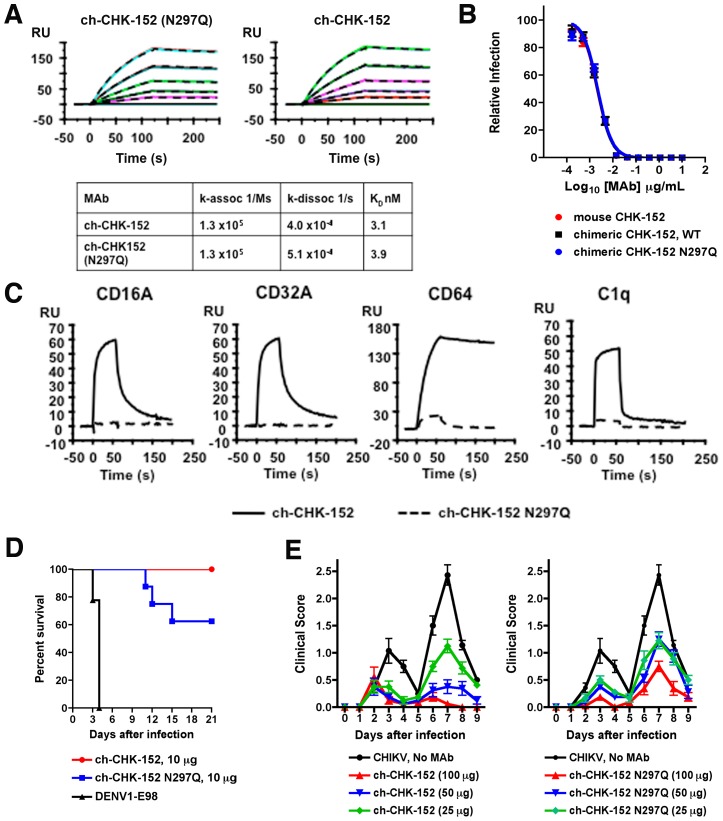
The effector functions of CHK-152 contribute to protection *in vivo*. **A**. Comparison of binding of ch-CHK-152 and agylocsyl ch-CHK-152 N297Q to pE2-E1, as measured by surface plasmon resonance. A single representative sensogram is shown for each MAb. The experimental curves (*colored lines*) were fit using a 1∶1 Langmuir analysis (*dashed lines*), after double referencing, to determine the kinetic parameters presented in the Table immediately below. **B**. Comparison of neutralizing activity of murine CHK-152, ch-CHK-152, and ch-CHK-152 N297Q, as measured by FRNT on Vero cells. **C**. Comparison of binding of ch-CHK-152 and ch-CHK-152 N297Q to FcγR (CD16A, 500 nM; CD32A, 100 nM; and CD64, 100 nM) or C1q (50 nM), as measured by surface plasmon resonance. **D**. Comparison of pre-exposure protective activity of ch-CHK-152 and ch-CHK-152 N297Q. *Ifnar* ^−/−^ mice were administered via an i.p. injection 10 µg of ch-CHK-152 and ch-CHK-152 N297Q one day before infection with 10 FFU of CHIKV-LR via a s.c. route. Mice were monitored for survival for 21 days after infection. The survival curves were constructed from data of at least two independent experiments and the number of animals for each antibody ranged from 8 to 10 per group. ch-CHK-152 provided statistically greater protection than ch-CHK-152 N297Q (*P*<0.05). **E**. Five week-old WT C57BL/6 mice were infected with 100 PFU of CHIKV in the left rear footpad and either sham-treated, or treated with 100, 50, or 25 µg of ch-CHK-152 (*left panel*) or ch-CHK-152 N297Q (*right panel*) at 18 hours post infection. Mice were scored daily for virus-induced footpad swelling, where a score of 0 = no swelling, 1 = mild swelling where the top of the foot is slightly raised, 2 = moderate swelling with the entire top of foot raised, and 3 = severe swelling involving both the top and bottom of the foot. Scores are the mean values for 7 to 8 mice per treatment group and are representative of three independent experiments. Ch-CHK-152 mediated protection was significantly greater than ch-CHK-152 N297Q on days 7, 8, and 9 post infection for the 100 µg antibody dose, and at day 7 post infection for the 50 µg dose, as determined by the Kruskal-Wallace test with Bonferroni correction (*P*<0.05). No statistically significant differences between ch-CHK-152 and ch-CHK-152 N297Q were observed with the 25 µg dose. Of note, we observed a reproducible decrease in clinical score on day 5 in many animals. This reflects the biphasic pattern of swelling: during the first 3 to 4 days, swelling is due to edema, whereas after day 5, it is due to inflammatory cell infiltration into the foot and ankle.

To define additional mechanisms by which our most strongly protective MAb (CHK-152) conferred protection *in vivo*, we generated a chimeric mouse-human CHK-152 (ch-CHK-152) as well as an aglycosyl variant (ch-CHK-152 N297Q) that lacks the ability to engage C1q or Fc-γ receptors; this mutation does not affect the ability to bind the neonatal Fc receptor (FcRn) or half-life of antibody in mouse serum [39]. The affinity of ch-CHK-152 and ch-CHK-152 N297Q binding to purified pE2-E1 was measured by surface plasmon resonance (SPR) and compared to the parent murine MAb. Notably, ch-CHK-152, ch-CHK-152 N297Q, and the murine CHK-152 all had similar affinity (*K_D_* of 3 to 4 nM) (**Fig. 4A** and data not shown) and neutralizing activity in cell culture (**Fig. 4B**). As expected, ch-CHK-152 N297Q failed to bind efficiently to soluble Fc-γ receptors or C1q (**Fig. 4C**).

We transferred ch-CHK-152 and ch-CHK152 N297Q to *Ifnar* ^−/−^ mice prior to infection. Although high doses (100 µg) of ch-CHK-152 and ch-CHK-152 N297Q provided similar protection against CHIKV infection (data not shown), lower doses (10 µg) of the aglycosyl variant were less protective; whereas 62% of the mice receiving ch-CHK152 N297Q survived, all *Ifnar* ^−/−^ mice given ch-CHK-152 MAb remained alive (**Fig. 4D**, *P*<0.05). When parallel studies were performed with WT C57BL/6 mice and MAb was administered 18 hours after infection, ch-CHK-152 N297Q also provided less protection against arthritis compared to ch-CHK-152 (**Fig. 4E**). These data suggest that the Fc effector interactions contribute to the potency of CHK-152 in mice.

### Humanization of CHK-152

We humanized CHK-152 as a first step towards a MAb therapeutic (see **Text S1**). The affinity for pE2-E1 and neutralizing activity of the hu-CHK-152 were similar to mouse CHK-152 (**Fig. S4A and B**). Hu-CHK-152 also protected *Ifnar* ^−/−^ mice (*P*>0.0001) when a single dose (10 or 100 µg) was administered one day before infection (**Fig. S4C**).

### Therapeutic studies

**Figure 5:**
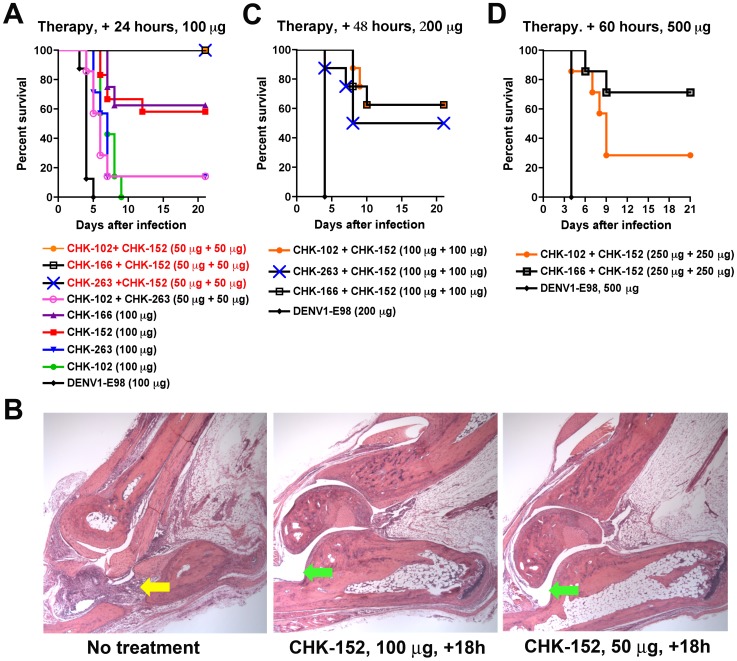
Therapeutic efficacy of anti-CHIKV MAbs. **A**. *Ifnar* ^−/−^ mice were passively transferred via an i.p. injection 100 µg of DENV1-E98, CHK-102, CHK-152, CHK-166, or CHK-263 or 50 µg each of CHK-102+CHK-152, CHK-166+CHK-152, CHK-263+CHK-152, or CHK-102+CHK-263 at 24 hours after CHIKV infection. **B**. Five week-old WT C57BL/6 mice were infected with 100 PFU of CHIKV in the footpad and either sham-treated, or treated with 100 or 50 µg of CHK-152 at 18 hours post infection. Virus induced pathology in the foot and ankle joint was assessed by histopathological analysis at day 10 post-infection. (*Left*) CHIKV-infected, sham-treated, (*middle*) CHIKV-infected, CHK-152 (100 µg) treated at +18 hours, and (*right*) CHIKV-infected, CHK-152 (50 µg) treated at +18 hours. Shown are representative images after hematoxylin and eosin staining from 3 mice per group at 100× magnification. Yellow and green arrows indicate regions of inflammation or normal joints, respectively. **C**. *Ifnar* ^−/−^ mice were passively transferred via an i.p. injection 200 µg of DENV1-E98 or 100 µg each of CHK-102+CHK-152, CHK-166+CHK-152, or CHK-263+CHK-152 at 48 hours after CHIKV infection. **D**. *Ifnar* ^−/−^ mice were passively transferred via an i.p. injection 500 µg of DENV1-E98 or 250 µg each of CHK-102+CHK-152 or CHK-166+CHK-152 at 60 hours after CHIKV infection. For **A**, **C**, and **D** the survival curves were constructed from data of at least two independent experiments. The number of animals for each antibody ranged from 8 to 10 per group, with the exception of CHK-102+CHK-263, which was performed with 7 mice only. Statistically significant differences in protection compared to DENV1-E98 are described in the text.

To define the therapeutic potential of our most protective MAbs, a single dose (100 µg) was administered to *Ifnar* ^−/−^ mice 24 hours after CHIKV infection (**Fig. 5A**). Whereas CHK-152 and 166 protected 58% and 63% of mice from death, respectively (*P*<0.0001), CHK-263 and CHK-102 had less activity although both MAbs increased the median survival time (7 days versus 4 days with the control DENV1-E98 MAb, *P*<0.0006). Administration of CHK-152 at 12 or 18 hours post infection also protected WT mice from CHIKV-induced swelling and arthritis (**Fig. 5B** **and** **Fig. 4E**).

We next tested the activity of combinations of the most protective neutralizing MAbs in *Ifnar* ^−/−^ mice. Remarkably, administration of 50 µg each (100 µg total dose) of CHK-102+CHK-152, CHK-263+CHK-152, or CHK-166+CHK-152 at 24 hours post infection completely prevented mortality in all animals (**Fig. 5A**, *P*<0.0001 for MAb combinations). This observation was not true for all MAb combinations, as administration of 50 µg each of CHK-102+CHK-263 provided substantially less protection with a 14% survival rate. We then performed a more stringent test in which 100 µg each (200 µg total) of our most protective combinations was delivered as a single dose at 48 hours post-infection (**Fig. 5C**). Treatment with CHK-102+CHK-152 or CHK-166+CHK-152 protected 62% of the *Ifnar* ^−/−^ mice (*P*<0.003) and the combination of CHK-263+CHK-152 functioned almost as well, with 50% of animals surviving (*P*<0.03). To define the limits of protection in *Ifnar* ^−/−^ mice, which all succumb to CHIKV between days 3 and 4, therapy was initiated at 60 and 72 hours after infection. At 60 hours after infection, *Ifnar* ^−/−^ mice receiving 250 µg each of CHK-102+CHK-152 or CHK-166+CHK-152 had survival rates of 28 and 71%, respectively (**Fig. 5D**, *P* = 0.03 and *P* = 0.004). Nonetheless, when combination therapy was given at 72 hours after infection, a time when overt disease was present, no survival benefit was conferred. Thus, combination MAb therapy is superior to monotherapy in protecting against lethal CHIKV infection in highly immunocompromised mice.

### Functional interaction of MAbs

To begin to understand the basis for enhanced *in vivo* activity, we assessed whether CHK-152 and selected MAbs could bind simultaneously to the CHIKV virion. We developed a competition ELISA in which virions were captured by a mouse MAb (CHK-65), and then incubated with increasing concentrations of CHK-102, CHK-152, CHK-166, or CHK-263 mouse MAbs. After washing, hu-CHK-152 MAb was added, and binding was assessed. While pre-bound mouse CHK-152 competed against hu-CHK-152 binding as expected, CHK-102, CHK-166, and CHK-263 minimally competed hu-CHK-152 binding (**Fig. S5A**), suggesting their epitopes largely were distinct. However, addition of CHK-102, CHK-166, or CHK-263 failed to augment the inhibitory activity of CHK-152 when neutralization was measured in cell culture (**Fig. S5B**), as no synergy was observed.

### Neutralization escape mutants

**Figure 6:**
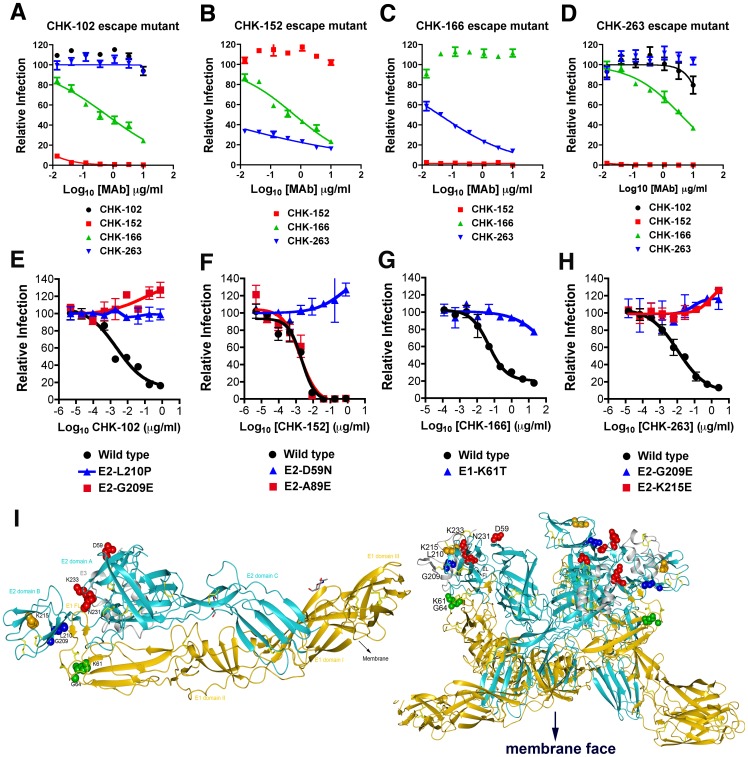
Characterization and mapping of neutralization escape mutants. **A–D**. FRNT assay with bulk virus obtained after three to six passages under selection of (**A**) CHK-102, (**B**) CHK-152, (**C**) CHK-166, or (**D**) CHK-263 on Vero cells. Bulk virus also was tested for infectivity in the presence of the non-selecting MAbs. Results are representative of two to three independent experiments performed in triplicate. **E–H**. Confirmation of resistant phenotype with SFV-CHIKV-GFP containing the indicated single engineered point mutations. Serial dilutions of MAb were incubated with chimeric SFV-CHIKV virus (WT or mutant stocks) for one hour at room temperature. MAb-virus complexes were added to Vero cells plated in 96-well plates and incubated at 37°C. After 8 hours cells were trypsinized, fixed, and the number of GFP-positive, infected cells was assessed by flow cytometry. Curves are representative of 2 to 3 independent experiments. **I**. Epitope mapping of anti-CHIKV MAbs on the crystal structure of the mature envelope glycoprotein complex (PDB code 3N44). (*Left*) The domains on E2 (cyan) and E1 (gold) are indicated, and the fusion loop on E1 (E1 FL) is delineated. Amino acid residues of neutralizing MAbs were determined by escape selection, sequencing, and reverse genetic confirmation. CHK-102 and CHK-263 recognize the B domain on E2, CHK-152 recognizes a residue on the wings of the A domain on E2, and CHK-166 recognizes an amino acid in domain II of E1 proximal to the conserved fusion loop. (*Right*) The mature envelope glycoprotein docked onto the trimer conformation (PDB code 2XFB) that is present on the virion. E3, E2, and E1 and the escape residues are colored as in the left panel. Neutralization escape residues are readily accessible on the top of the trimer, distal to the viral membrane.

To identify epitopes targeted by the therapeutic MAbs, we generated escape mutants in cell culture. After sequential virus passage under CHK-102, CHK-152, CHK-166, or CHK-263 selection, CHIKV became resistant to neutralization by these MAbs (**Fig. 6A–D**). We assessed whether the escape variants generated in the presence of one MAb remained sensitive to neutralization by the other MAbs. The CHK-152 escape variant was neutralized efficiently by CHK-102, CHK-166, and CHK-263 (**Fig. 6B** **, Table S2** in **Text S1**, and data not shown), and analogously the CHK-166 escape variant was inhibited by CHK-102, CHK-152, and CHK-263 (**Fig. 6C**, and data not shown). In contrast, CHK-102 and CHK-263 escape variants reciprocally were resistant, suggesting their epitopes were the same or overlapping (**Fig. 6A and D**); however, CHK-102 and CHK-263 escape variants remained sensitive to neutralization by CHK-152 and CHK-166. Notably, selection with combinations of MAbs (e.g., CHK-102+CHK-152) failed to produce escape variants despite several independent attempts (data not shown).

To identify the mutations that conferred resistance, we sequenced plaque-purified escape variants (**Table 2**, *top*). Six of eight sequences from CHK-102 escape variants contained an L210P mutation in the E2 protein; the remaining two sequences had a G209E mutation in E2. For CHK-152 resistant variants, all sequences (9 of 9) contained a D59N mutation in E2 and two contained a second A89E substitution in E2. For CHK-263, 3 of 4 escape variants had a K215E change in E2, whereas 1 of 4 had mutations in E2 at G209E. All escape variants (14 of 14) of CHK-166 had a single K61T mutation in the E1 protein.

**Table 2:** *In vitro* selection of viruses resistant to MAb neutralization.

MAb	Mutation^a^	# of plaque picks
CHK-102	E2: L210P	6 of 8
CHK-102	E2: G209E	2 of 8
CHK-152	E2: D59N	9 of 9
CHK-152	E2: A89E^b^	2 of 9
CHK-166	E1: K61T	14 of 14
CHK-263	E2: K215E	3 of 4
CHK-263	E2: G209E	1 of 4

a *In vitro* selection for neutralization escape variants was performed by passaging CHIKV-LR in the presence of 25 µg/ml of the indicated MAbs. Resistant virus was isolated at passage 3 (CHK-102, CHK-152, and CHK-263) or passage 6 (CHK-166), plaque purified, and sequenced.

b The A89E mutant was identified after sequencing of CHK-152 escape mutants in cell culture, but was determined to be insignificant for CHK-152 neutralization by reverse genetic analysis (see **Fig. 6F**).

To verify the amino acid changes that conferred MAb resistance *in vitro*, we introduced several of these substitutions into a chimeric SFV-GFP-CHIKV cDNA comprised of SFV non-structural genes, a GFP reporter gene, and the CHIKV structural genes (T. Lin, K. Dowd, and T. Pierson, unpublished results). Parental and SFV-GFP-CHIKV with single amino acid mutations were analyzed for neutralization by CHK-102, CHK-152, CHK-166, and CHK-263 (**Fig. 6E–H**). Consistent with our sequencing results, viruses encoding mutations in E2-G209 and E2-L210 were resistant to CHK-102, changes in E2-D59 conferred resistance to CHK-152, substitutions in E1-K61 resulted in resistance to CHK-166, and mutation of E2-G209 and E2-K215 caused resistance to CHK-263. However, introduction of E2-A89E (which was present in 2 of 9 clones) failed to affect the neutralizing activity of CHK-152.

In addition to selecting escape variants in cell culture, we harvested organs from the few mice that became ill after infection despite single MAb treatment (**Table 3**, *bottom*). In these moribund *Ifnar* ^−/−^ mice, CHIKV was present in the brain and muscle but absent from the spleen or liver (data not shown). This *in vivo*-derived virus was tested for MAb resistance and sequenced. For mice receiving a 10 µg dose of CHK-102 as prophylaxis, resistant variants with a L210P mutation in E2 were obtained. For mice receiving CHK-263 or CHK-102 at 24 hours post infection, resistant viruses with a G209E mutation in E2 were identified. None of the animals that were pre-treated with 10 µg of CHK-166 developed escape mutants, as the virus harvested from all 3 mice tested retained sensitivity to CHK-166 (data not shown). However, in one animal receiving CHK-166 at 24 hours post infection, a single resistant virus with a G64S substitution in the E1 gene was recovered (**Fig. S6**). For mice receiving a 10 µg dose of hu-CHK-152 as prophylaxis, partially resistant viruses with N231D and K233E mutations in E2 were isolated and confirmed by reverse genetics using the chimeric SFV-GFP-CHIKV infectious clone (**Fig. S7**). In comparison, when CHK-152 was given as a therapeutic, a single mutation at D59N in E2 was obtained in 4 of the 5 mice tested, with a K233T mutation in virus from the remaining animal. For animals treated at 48 hours with combination MAb therapy, all recovered viruses remained sensitive to CHK-152 yet showed partial resistance to CHK-102 or CHK-166 (**Fig. S8**). Mutations in E2 (N332I, CHK-166+CHK-152) were identified. Comparison of 140 available E1 and E2 sequences from historical and circulating CHIKV strains in a public database (http://www.viprbrc.org/) revealed nearly complete conservation of the residues in which escape mutants were selected: E1-K61, 100%; E1-G64, 100%; E2-D59, 100%; E2-G209, 100%; E2-L210, 99.3%; E2-K215, 100%; E2-N231, 100%; and E2-K233, 99.3%.

**Table 3:** *In vivo* selection of viruses resistant to MAb neutralization.

MAb	Condition	EC50 (ng/ml) (Parent→mutant)	Mutation^a^
CHK-102	−24 h, 10 µg	11**→**>10,000	E2: L210P
CHK-102	+24 h, 100 µg	11**→**>10,000	E2: G209E
CHK-152	−24 h, 10 µg	2**→**10,000	E2: N231D
			E2: K233E
CHK-152	−24 h, 10 µg	2**→**3,000	E2: K233E
CHK-152	+24 h, 100 µg	2**→**>10,000	E2: D59N
CHK-152	+24 h, 100 µg	2**→**>10,000	E2: K233T
CHK-166	+24 h, 100 µg	170**→**>10,000	E1: G64S
CHK-263	+24 h, 100 µg	5**→**>10,000	E2: G209E
CHK-166+CHK-152	+48 h, 250 µg	CHK-166: 170→540	E2: N332I
		CHK-152: 2→2.6	

a *In vivo* selection for resistant virus was performed by administering the indicated individual or combinations of MAbs before (−24 hours) or after (+24 or 48 hours) CHIKV-LR infection. Resistant virus was isolated directly from tissues (leg and brain), and cDNA was prepared by reverse transcription and sequenced. The change in neutralizing activity of the bulk virus recovered from tissue is highlighted by the differences in EC50 values.

To define spatially the location of the amino acids that conferred resistance to our highly protective MAbs, these residues were mapped onto the existing CHIKV protein crystal structures [10] (**Fig. 6I**, *left*). Amino acids that conferred neutralization escape to CHK-102 and CHK-263 were located in the B domain of E2. The residues that modulated CHK-152 neutralization mapped to the A domain of E2. In contrast, CHK-166 recognized amino acids on DII of E1, adjacent to the fusion loop. All amino acids that conferred neutralization escape appear solvent accessible and highly exposed when docked onto the E2-E1 spike (**Fig. 6I**, *right*).

## Discussion

We set out to identify MAbs with the greatest therapeutic activity against CHIKV in mice as a first step toward generating an immunotherapy for humans. Thirty-six MAbs with neutralizing activity against CHIKV-LR were identified, the majority of which also inhibited infection of strains corresponding to the two heterologous CHIKV genotypes. Although all fourteen of the selected anti-CHIKV MAbs improved outcome in vulnerable *Ifnar* ^−/−^ mice, only four of these (CHK-102, CHK-152, CHK-166, and CHK-263) completely prevented lethality when administered as prophylaxis. CHK-152 provided the greatest benefit as post-exposure therapy, although by itself, the window of treatment activity was limited in the *Ifnar* ^−/−^ mouse model. While addition of a second MAb (CHK-102, CHK-166, or CHK-263) failed to enhance CHK-152 neutralization *in vitro*, it limited the development of viral resistance *in vitro* and *in vivo*. Remarkably, combinations of CHK-102+CHK-152 or CHK-166+CHK-152 protected *Ifnar* ^−/−^ mice against mortality even when a single dose was administered 24 to 36 hours prior to the death of untreated or isotype control MAb-treated animals.

In comparison to the highly therapeutic activity of 0.5 mg of CHK-152+CHK-166, a single 25 mg dose of immune IgG purified from a convalescent human subject protected only 50% of *Ifnar* ^−/−^ mice when administered 24 hours after CHIKV infection [26]. The administered dose of neutralizing antibody likely is critical to post-exposure treatment of CHIKV infection because of the high viral burden [14], [16], [17], [40]. A high viral load impacts therapeutic activity of antibodies as it (a) increases the chance for pre-existing or selected resistant variants to emerge through quasispecies [28], [41]; and (b) results in a low relative fractional occupancy of binding to any individual virion, which allows antibodies recognizing key epitopes to fall below their stoichiometric threshold of neutralization [42]. Although there is extensive literature on the protective efficacy of MAbs or immune sera against alphavirus infection [18]–[25], no prior study has demonstrated reduced CHIKV-induced mortality with MAbs. Although a recent study showed that combination post-exposure therapy with two human anti-CHIKV MAbs (5F10 and 8B10, 250 µg each at +8 h) prolonged survival of AG129 (*Ifnar* ^−/−^×*Ifngr* ^−/−^) mice by ten days, they failed to prevent lethal infection [29]; the basis of this treatment failure remains unclear but could reflect the lower neutralizing potency of the MAbs (compared to CHK-152), rapid emergence of resistant mutants, or the relative susceptibility of the immunocompromised mouse host. In comparison, a neutralizing MAb (UM 5.1) administered two days after SFV infection completely protected immunocompetent BALB/c mice [43].

Why were some combinations of two MAbs effective *in vivo*? (a) Pairs of MAbs may show neutral, additive, or synergistic effects on neutralization. Positive antiviral effects could occur through cooperative binding or by trapping CHIKV in conformations that makes it less competent to bind a receptor or fuse with host membranes. Nonetheless, when we added increasing concentrations of CHK-102, CHK-166, or CHK-263 to CHK-152, we failed to observe synergy. (b) Certain MAb combinations could prevent the emergence of resistance due to the low frequency of two escape mutations occurring simultaneously in a single replication cycle. Although we could readily select for neutralization escape against a single MAb *in vitro* and *in vivo*, we failed to isolate resistant mutants against CHK-152 when two MAbs (e.g., CHK-102+CHK-152) were combined. However, some viruses from moribund animals treated with combination MAb therapy showed reduced sensitivity (up to 200-fold) to the other MAb (e.g., CHK-102) in the pair. In comparison, when mice were treated with a combination of 50 µg each of CHK-102+CHK-263, we failed to observe the same survival benefit that was conferred by the combinations of CHK-102, CHK-166, or CHK-263 with CHK-152. Since CHK-102 and CHK-263 appear to share overlapping footprints on domain B of E2, this particular MAb combination may fail to prevent the rapid emergence of escape mutants relative to others targeting distinct epitopes on E1 and E2 proteins. (c) Combinations of MAbs could select for resistant viruses that have reduced fitness [44], and thus are less pathogenic *in vivo*. Virulence studies with CHIKV encoding selected single and double mutations are planned to evaluate this possibility.

We localized the epitopes of our four highly protective MAbs using neutralization escape selection, sequencing, and reverse genetics. CHK-152, which blocked viral fusion, mapped to the wings of the A domain on E2, a result that we recently confirmed by cryo-electron microscopic analysis of CHK-152 Fab-virus particle complexes [45]. This epitope also was identified as a recognition site for neutralizing MAbs against VEEV [46] and SINV [47]. CHK-166, which was the least neutralizing (EC50 of ∼100 ng/ml) of our highly protective MAbs mapped to an epitope in domain II of the E1 protein, adjacent to the highly conserved fusion loop. While anti-E1 MAbs against SINV and VEEV that protect or neutralize infection have been described [46], [48], [49], none have been characterized against CHIKV. A neutralizing human MAb (8B10) against CHIKV was reported with possible reactivity against E1, although further analysis revealed that it bound to the E1/E2 heterodimer [27], [28]. CHK-102 and CHK-263 mapped to residues within the B domain on E2. A related epitope also was identified in mapping studies of strongly neutralizing antibodies against Ross River virus [50], SINV [51], [52], VEEV [46], [53], [54], and CHIKV [10], [28]. The B domain on E2 comprises an important antigenic domain that is under selective pressure for antibody neutralization [41]. It serves as a cap to the fusion loop on E1 and because of its location at the tip of the heterodimeric spike [10], [11] may contribute to attachment of cellular receptors.

In summary, we identified combinations of MAb pairs that were highly effective as post-exposure therapeutic agents. These findings are consistent with recent studies showing enhanced post-exposure efficacy of MAb combinations against Ebola [55], influenza A [56] and rabies [57] viruses. Our most promising pair of MAbs mapped to distinct epitopes, limited the generation of resistance, blocked multiple stages of the viral entry pathway, and protected *Ifnar* ^−/−^ mice against mortality even when administered 60 hours after infection. CHK-152 was humanized as a first step towards a possible therapeutic for humans and demonstrated similar efficacy compared to the parent murine MAb. Tailored combinations of potently neutralizing MAbs show promise to prevent or treat infection by CHIKV, and likely other pathogenic alphaviruses in humans. Ultimately, a more detailed kinetic analysis of CHIKV infection in humans and determination of a treatment window relative to symptom onset is warranted to establish whether combination MAb therapy can prevent or mitigate acute or chronic and persistent infection and joint disease.

## Methods

### Cells and viruses

Vero, Vero76 (ATCC), BHK21-15, and NIH 3T3 mouse fibroblast cells were cultured in Dulbecco's modified Eagle medium (DMEM) supplemented with 5% or 15% (for 3T3 cells) fetal bovine serum (FBS) (Omega Scientific). C6/36 *Aedes albopictus* cells were grown in Leibovitz-15 medium supplemented with 10% FBS at 27°C. The infectious clones of CHIKV La Reunion 2006 OPY-1 (strain 142, CHIKV-LR) and CHIKV-GFP (strain 145) were gifts from S. Higgs (Manhattan, KS) [58]. CHIKV-RSU1 and CHIKV-IbH35 were gifts of R. Tesh, (Galveston, TX). Infection studies of WT mice used the SL15649 strain of CHIKV, which was generated from an infectious clone [17]. The S27 African prototype CHIKV strain was a gift from Dr. S. Günther (Bernhard-Nocht-Institute for Tropical Medicine, Germany) and isolated from a patient in Tanzania in 1953.

### Ethics statement

This study was carried out in strict accordance with the recommendations in the Guide for the Care and Use of Laboratory Animals of the National Institutes of Health. The protocols were approved by the Institutional Animal Care and Use Committee at the Washington University School of Medicine (Assurance Number: A3381-01) and the University of North Carolina (A3410-04). Dissections and footpad injections were performed under anesthesia that was induced and maintained with ketamine hydrochloride and xylazine, and all efforts were made to minimize suffering.

### Generation of chimeric SFV-CHIKV

Chimeric SFV-CHIKV virus was generated by complementation of a double sub-genomic DNA-launched SFV replicon “backbone” plasmid (pSFV-GFP-BB) with the structural genes of CHIKV as described recently for WNV [59]. The vectors and methods will be described in detail elsewhere (TY Lin, K. Dowd, and T. Pierson, in preparation). To generate SFV-CHIKV, a DNA fragment encoding WT or mutant CHIKV structural genes was ligated into the pSFV-GFP-BB plasmid and transfected directly into HEK-293T cells using Lipofectamine LTX. The source of CHIKV structural genes was a sub-cloning vector pCHIKV-struct: mutations were introduced into this vector using site-directed mutagenesis and fully sequenced. Virus was harvested at 48, 72, or 96 hours after transfection, filtered, and stored at −80°C.

### CHIKV protein

The CHIKV E2 ectodomain (residues S1-E361) and pE2-E1 (E3-E2-E1: residues S1-R64 of E3, S1-E161 of E2, and Y1-Q411 of E1 including a (GGGS)_4_ polylinker between E2 and E1) of the CHIKV-LR strain were amplified from the infectious cDNA clone using high-fidelity Phusion PCR (Thermo Scientific). The E2 ectodomain was cloned into pET21a, expressed in *E. coli*, and purified using an oxidative refolding protocol followed by size-exclusion column purification using fast protein liquid chromatography [60]. pE2-E1 was cloned into the mammalian expression vector pHLsec (Invitrogen) with a C-terminal octa-histidine tag and modified to express the Epstein–Barr virus EBNA-1 protein for enhanced protein expression. pE2-E1 was expressed in serum-free HEK-293F suspension cells and purified by Ni-NTA agarose affinity (Qiagen) and Superdex 200 gel filtration chromatography.

### MAb generation

*Irf7* ^−/−^ mice were infected and boosted with 10^4^ PFU of CHIKV-LR and, depending on the experiment, given a final intravenous (i.v.) boost with CHIKV virus-like particles [30], 25 µg of E2 protein, or 2×10^5^ PFU of CHIKV-LR three days prior to fusion with myeloma cells. Hybridomas secreting antibodies that reacted with CHIKV-GFP-infected BHK21-15 cells were identified by flow cytometry and cloned by limiting dilution. MAbs were isotyped by ELISA (Pierce), adapted for growth under serum-free conditions, and purified by protein G affinity and size exclusion chromatography. All MAbs were screened initially with a single endpoint neutralization assay using neat hybridoma supernatant (∼10 µg/ml), which was incubated with 100 FFU of CHIKV-LR for one hour at 37°C. MAb-virus complexes were added to BHK21-15 cell monolayers in 6-well plates. After 90 minutes, cells were overlaid with 1% (w/v) agarose in Modified Eagle Media (MEM) supplemented with 4% FBS. Plates were fixed with 10% formaldehyde in PBS 48 hours later, stained with crystal violet, and plaques were counted. The V_H_ and V_L_ sequence of neutralizing MAbs CHK-102, CHK-152, CHK-166, and CHK-263 were amplified from hybridoma cell RNA by a 5′ RACE procedure **Table S3** in **Text S1**).

### Chimerization of MAbs

The generation of a chimeric mouse-human CHK-9 and CHK-152 with mouse V_H_ and V_L_ and human IgG1 constant regions was performed as described previously [60]. A point mutation that abolishes FcγR and C1q binding (N297Q) was introduced by QuikChange mutagenesis (Stratagene). Recombinant antibodies were produced after transfection of HEK-293T cells, harvesting of supernatant, and purification by protein A affinity chromatography.

### Infection of mice

#### (a) Immunocompromised mice

*Ifnar* ^−/−^ mice were bred in pathogen-free animal facilities of the Washington University School of Medicine and infection experiments were performed in A-BSL3 facilities with the approval of the Washington University Animal Studies Committee. For prophylaxis studies, MAbs were administered by i.p. injection to 6 to 8 week-old *Ifnar* ^−/−^ mice one day prior to s.c. infection in the footpad with 10 FFU of CHIKV-LR. For therapeutic studies, 10 FFU of CHIKV-LR was delivered 24, 48, 60, or 72 hours prior to administration of a single dose of individual or combinations of MAbs. To monitor viral burden *in vivo*, mice were treated with a single 100 µg dose of anti-CHK or isotype control MAb one day before infection with 10 FFU of CHIKV-LR. Animals were sacrificed two days later for virological analysis. After extensive perfusion with PBS, organs were harvested, weighed, homogenized and virus was titered by focus-forming assay.

#### (b) Immunocompetent mice

Four to six week-old C57BL/6 mice were infected s.c. in the footpad with 100 PFU of CHIKV SL15649 in 10 µl of PBS as described previously [17]. Some animals received 100 µg of MAb in 500 µl of PBS via an i.p. route before or after infection. Mice were monitored daily for footpad swelling. At 10 days after infection, mice were sacrificed and sections prepared from decalcified hind limbs [17] for histopathological analysis. All CHIKV studies with WT mice were performed under A-BSL-3 conditions and in accordance with approved protocols following University of North Carolina guidelines.

### Neutralization assays

Serial dilutions of MAb were incubated with 100 FFU of CHIKV for one hour at 37°C. MAb-virus complexes were added to cells in 96-well plates. After 90 minutes, cells were overlaid with 1% (w/v) methylcellulose in Modified Eagle Media (MEM) supplemented with 4% FBS. Plates were harvested 18 to 24 hours later, and fixed with 1% PFA in PBS. The plates were incubated sequentially with 500 ng/ml of ch-CHK-9 and horseradish peroxidase (HRP)-conjugated goat anti-human IgG in PBS supplemented with 0.1% saponin and 0.1% BSA. CHIKV-infected foci were visualized using TrueBlue peroxidase substrate (KPL) and quantitated on an ImmunoSpot 5.0.37 macroanalyzer (Cellular Technologies Ltd). Non-linear regression analysis was performed, and EC50 values were calculated after comparison to wells infected with CHIKV in the absence of antibody.

### Pre- and post-attachment neutralization assays

96-well tissue culture plates were coated with 100 µl of poly-L lysine and seeded with 3×10^4^ Vero cells/well overnight. For pre-attachment assays, dilutions of MAb were prepared at 4°C in DMEM with 2% FBS and pre-incubated with 100 FFU of CHIKV-LR for one hour at 4°C. MAb-virus complexes were added to pre-chilled Vero cells for one hour at 4°C. Non-adsorbed virus was removed with three washes of DMEM and adsorbed virus was allowed to internalize during a 37°C incubation for 15 minutes. Cells were overlaid with 1% (w/v) methylcellulose in MEM supplemented with 4% FBS. The post-attachment assay was performed similarly, except that an equivalent amount of CHIKV was adsorbed first onto Vero cells for one hour at 4°C. After removing free virus, dilutions of MAb were added to the virus-adsorbed cells for one hour at 4°C. Virus was allowed to internalize and cells were overlaid with methylcellulose as described above. Nineteen hours later, the plates were harvested and analyzed for antigen-specific foci as described above.

### Fusion inhibition assays

#### (a) Fusion from without assay

Virus fusion with the plasma membrane was assessed using a fusion from without (FFWO) assay [36]. Vero cells were seeded in 96-well plates, washed once with Binding medium (RPMI 1640, 0.2% BSA, 10 mM HEPES pH 7.4, and 20 mM NH_4_Cl) at 4°C, and incubated for 15 minutes at 4°C. CHIKV-LR (MOI of 15) was prepared in Binding medium and added to cells for one hour at 4°C, and then free virus was removed. Subsequently, DMEM containing 2% FBS with or without CHIKV-specific or control MAbs (50 µg/ml) was added to cells for one hour at 4°C. FFWO was induced by the addition of pre-warmed fusion media (RPMI 1640, 0.2% BSA, 10 mM HEPES, and 30 mM succinic acid at pH 5.5) for two minutes at 37°C. In parallel wells, control media (RPMI 1640, 0.2% BSA, 10 mM HEPES at pH 7.4) was added for 2 minutes at 37°C to ensure that infection occurred only through pH-dependent plasma membrane fusion. Medium was removed and cells were incubated in DMEM supplemented with 5% FBS, 10 mM HEPES, and 20 mM NH_4_Cl (pH 7.4); NH_4_Cl prevented secondary infection through endosomal fusion pathways. Cells were detached 14 hours later, fixed with 1% PFA in PBS for 8 minutes, and permeabilized with 0.1% (w/v) saponin detergent solution. Cells were incubated sequentially with ch-CHK-9 and Alexa 647 conjugated goat anti-human IgG secondary antibody (Invitrogen). Infection was evaluated on a FACSArray flow cytometer (Becton-Dickinson) and analyzed using FlowJo software.

#### (b) Liposomal fusion assay

Pyrene-labeled CHIKV (S27 African strain) was recovered from supernatants of infected Vero76 cells cultured for 48 hours in the presence of 15 µg/ml 16-(1-pyrenyl)hexadecanoic acid (Invitrogen) as described [37]. Fusion of pyrene-labeled CHIKV with liposomes was monitored continuously in a Fluorolog 3–22 fluorometer (BFi Optilas), essentially as described [37]. Pyrene-labeled CHIKV and an excess of liposomes were mixed in a final volume of 665 µl in 5 mM HEPES, 150 mM NaCl, 0.1 mM EDTA, pH 7.4. Fusion was triggered by the addition of 35 µl 0.1 M MES, 0.2 M acetic acid, which achieved a pH of 4.7. For the antibody inhibition experiments, pyrene-labeled CHIKV was incubated with increasing concentrations of CHIKV-152 or isotype control IgG2a MAb (MAb 0031, R&D systems) for 10 minutes at 37°C prior to mixing with liposomes.

### SPR

The binding of human FcγR and C1q to ch-CHK-152 and ch-CHK-152 (N297Q) was analyzed by SPR using a BIAcore 3000 biosensor (GE Healthcare Life Sciences). MAbs were captured (∼900 RU) after flowing over immobilized F(ab)′2 fragments of goat anti-human F(ab)′2 specific IgG on a CM-5 sensor chip. Binding experiments were performed in HBS-EP buffer (10 mM Hepes, pH 7.4, 150 mM NaCl, 3 mM EDTA, and 0.005% P20 surfactant). Binding of CD16A and CD64 (as monomeric soluble FcγR), CD32A (as dimeric soluble FcγR-aglycosylated Fc fusion), and C1q (Sigma-Aldrich) was analyzed at a single concentration. The FcγR and C1q were injected for 60 sec at a flow rate of 30 µl/min then allowed to dissociate over 2 minutes. Affinity measurements of CHK-152 MAbs for pE2-E1 were performed by SPR in HBS-EP buffer. Ch-CHK-152, ch-CHK-152 N297Q, hu-CHK-152 and mouse CHK-152 were captured (∼300 RU) after flowing over immobilized F(ab)′2 fragments of goat anti-human or anti-mouse Fc specific IgG. Purified pE2-E1 was injected at concentrations of 0, 6.25, 12.5, 25, 50, and 100 nM, at a flow rate of 30 µl/min for 120 sec, and then allowed to dissociate over 2 minutes. Regeneration of capture surfaces was performed by pulse injection of 10 mM glycine pH 1.5. Binding curve at the zero concentration of pE2-E1 was subtracted from each experimental curve as a blank. Data were analyzed using BIAevaluation 4.1 software. Kinetic constants, k_a_ and k_d_, were estimated by global fitting analysis of the association/dissociation curves to the 1∶1 Langmuir interaction model.

### Escape mutant selection

CHIKV-LR (1.2×10^5^ FFU) was incubated with 25 µg/ml of MAbs for one hour at 37°C. Virus-MAb complexes were added to Vero cells and infection proceeded for 24 hours. At each passage, half of the supernatant was mixed (1∶1) with 50 µg/ml of the selection MAb for one hour at 37°C. These complexes were added to a new monolayer of Vero cells for 2 hours, and the procedure was repeated from 3 to 6 times depending on the selection MAb. Individual MAb-resistant viral plaques were picked and virus was grown in Vero cells in the presence of 10 µg/ml of MAb for 24 hours. RNA was isolated from cells using an RNeasy kit (Qiagen) and cDNA was made with random hexamers using the Superscript III Reverse Transcriptase kit (Invitrogen) and amplified by PCR with primers flanking the structural genes (**Table S4**). The PCR product was sequenced using ten overlapping primer sets (**Table S4**).

### Mapping of mutations onto the CHIKV p62-E1 crystal structure

Figures were prepared using the atomic coordinates of CHIKV pE2-E1 (RCSB accession number 3N44) using the program CCP4MG [61].

### Statistical analysis

For survival analysis, Kaplan-Meier survival curves were analyzed by the log-rank test. For growth kinetics and neutralization an unpaired T-test or analysis of variance was used to determine significance. These analyses were assessed using Prism software (GraphPad software). The protective effects of ch-CHK-152 versus ch-CHK-152 N297Q in wild type C57BL/6 mice were analyzed by the Kruskal-Wallace test with Bonferroni correction using the agricolae package of R (R Development Core Team, 2010. Foundation for Statistical Computing, Vienna, Austria).

## Supporting Information

Figure S1 **Screening of hybridoma supernatants for binding to CHIKV-infected cells.** Hybridoma supernatants were incubated with CHIKV-GFP infected BHK21 cells and tested for immunoreactivity by flow cytometry. Shown are examples of a negative control MAb (DENV3-E2), three ‘hits’ (later named as CHK-102, CHK-117, and CHK-130), and a negative supernatant (5E3). The y-axis shows GFP staining associated with the reporter gene that is translated from the subgenomic promoter of CHIKV, and the x-axis shows staining of the tested mouse MAb. Double-positive cells were considered ‘hits’ in the screen. The result is representative of many different MAbs performed in the original screen.
(TIF)

Figure S2 **Binding kinetics of CHK-MAbs to pE2-E1.** Binding curves and kinetic parameters of pE2-E1 binding to mouse CHK-102, CHK-152, CHK-166, and CHK-263 MAbs. A single representative sensogram is shown for each MAb. The experimental curves (*colored lines*) were fit using a 1∶1 Langmuir analysis (*dashed lines*), after double referencing, to determine the kinetic parameters presented immediately below.
(TIF)

Figure S3 **Pre- and post-attachment neutralization assays.** Vero cells were pre-chilled to 4°C and 100 FFU of CHIKV-LR was added to each well for one hour at 4°C. After extensive washing at 4°C, the indicated MAbs (CHK-48, CHK-65, CHK-95, CHK-112, CHK-124, CHK-142, CHK-155, CHK-175, CHK-84 and DENV1-E98) were added for one hour at 4°C, and then the FRNT protocol was completed (*black lines*, *Post*). In comparison, a standard pre-incubation FRNT with all steps performed at 4°C is shown for reference. Virus and MAb are incubated together for one hour at 4°C, prior to addition to cells (*red lines, Pre*). Data shown are representative of three experiments performed in duplicate with error bars representing standard deviation.
(TIF)

Figure S4 **Construction and efficacy of humanized CHK-152.** We amplified the cDNA encoding the heavy (VH) and light (VL) variable domains from the hybridoma cellular RNA and grafted the complementarity determining regions onto the human VH1-18 and human Vκ-L6 backbones. The resulting humanized VH and VL were combined with human γ1 and κ constant regions, fused to an IgG signal sequence, expressed in 293T cells and purified (data not shown). **A.** Binding curves and kinetic parameters of pE2-E1 binding to mouse CHK-152 and hu-CHK-152. A single representative sensogram is shown for each MAb. The experimental curves (*colored lines*) were fit using a 1∶1 Langmuir analysis (*dashed lines*), after double referencing, to determine the kinetic parameters presented in the Table immediately below. **B.** Neutralization studies with mouse CHK-152 and hu-CHK-152. Neutralizing activity was determined by FRNT assay on Vero cells. Samples were performed in duplicate and the experiment is one representative of three. **C.** Pre-exposure protective activity of hu-CHK-152. *Ifnar* ^−/−^ mice were passively transferred via an i.p. injection 10 or 100 µg of mouse hu-CHK-152 one day before CHIKV infection. Mice were monitored for survival for 21 days after infection. The survival curves were constructed from data of at least two independent experiments and the number of animals for each antibody ranged from 8 to 10 per group.
(TIF)

Figure S5 **Interaction of neutralizing MAbs.** **A.** Virion capture ELISA and competition of MAb binding. 96-well plates were coated with 5 µg/ml of CHK-65 MAb. Non-specific binding sites were blocked, and 3×10^6^ FFU of CHIKV 181-25 was captured. Subsequently, plates were incubated with the indicated anti-CHK mouse MAbs (CHK-102, CHK-152, CHK-166, or CHK-263) or controls (no MAb, PBS; irrelevant MAb, WNV E28) for one hour. After washing, plates were incubated sequentially with 125 ng/ml hu-CHK-152 and biotin-labeled goat anti-human secondary antibody. After washing and incubation with HRP-conjugated streptavidin, plates were developed and emission (450 nm) was read using an iMark microplate reader (Bio-Rad). Results are representative of three independent experiments, each performed in triplicate. **B.** Neutralizing activity of MAb combinations. Increasing concentrations of individual MAbs (CHK-102, CHK-152, CHK-166, and CHK-263) or combinations of MAbs (CHK-102+CHK-152, CHK 102+CHK-263, CHK-152+CHK-166, or CHK-152+CHK-263) were mixed with 100 FFU of CHIKV-LR for one 1 hour at 37°C and Vero cells were infected. Neutralization was determined by FFU assay. Data is representative of three independent experiments performed in duplicate.
(TIF)

Figure S6 **Selection of escape E1-G64S escape mutant** ***in vivo*** **against CHK-166.** *Ifnar* ^−/−^ mice were infected with CHIKV and 24 hours later administered a single 100 µg dose of CHK-166. Six days later, virus was recovered from the contralateral leg and brain from one moribund mouse and the structural genes were sequenced. Both viral isolates recovered showed a single point G64S mutation in the E1 gene. This isolate was tested for neutralization by CHK-102 (EC50 of 161 ng/ml), CHK-152 (EC50 of 2 ng/ml), CHK-166 (EC50>10,000 ng/ml) and CHK-263 (25 ng/ml). Data is the average of two independent experiments performed in triplicate.
(TIF)

Figure S7 **Confirmation of neutralization escape mutants selected** ***in vivo*** **.** Confirmation of resistant phenotype selected with CHK-152 *in vivo* using SFV-CHIKV-GFP containing the indicated single engineered point mutations. Serial dilutions of CHK-152, CHK-102, and CHK-263 were incubated with chimeric SFV-CHIKV virus (WT or mutant stocks) for one hour at room temperature. MAb-virus complexes were added to Vero cells plated in 96-well plates and incubated at 37°C. After 8 hours cells were trypsinized, fixed, and the number of GFP-positive infected cells was assessed by flow cytometry. Curves are representative of 2 independent experiments.
(TIF)

Figure S8 **Relative resistance of CHIKV recovered from mice after treatment with combination MAb therapy.** *Ifnar* ^−/−^ mice were infected with CHIKV and 48 hours later given a single dose of combination MAb (CHK-102+CHK-152 or CHK-166+CHK-152) therapy. Virus was recovered from the contralateral leg and/or brain from the few moribund mice and the structural genes were sequenced. Two viral isolates showed differences in neutralization patterns that corresponded to amino acid substitutions (see **Table 2**). Neutralization analysis of these viruses recovered from animals treated with (*left*) CHK-102 and CHK-152 or (*right*) CHK-166 and CHK-152 and tested against the respective MAbs. A comparison with the parent virus is shown. The curves are representative of two independent experiments performed in triplicate, and error bars indicate standard deviations.
(TIF)

Text S1 **Supplemental Methods and Tables S1–S4.**
(DOCX)
