# Supplementary figures and images for: Development of a Highly Protective Combination Monoclonal Antibody Therapy against Chikungunya Virus
Source: PLoS Pathog. 2013 Apr 18;9(4):e1003312. doi: 10.1371/journal.ppat.1003312 (PMC3630103; doi:10.1371/journal.ppat.1003312)

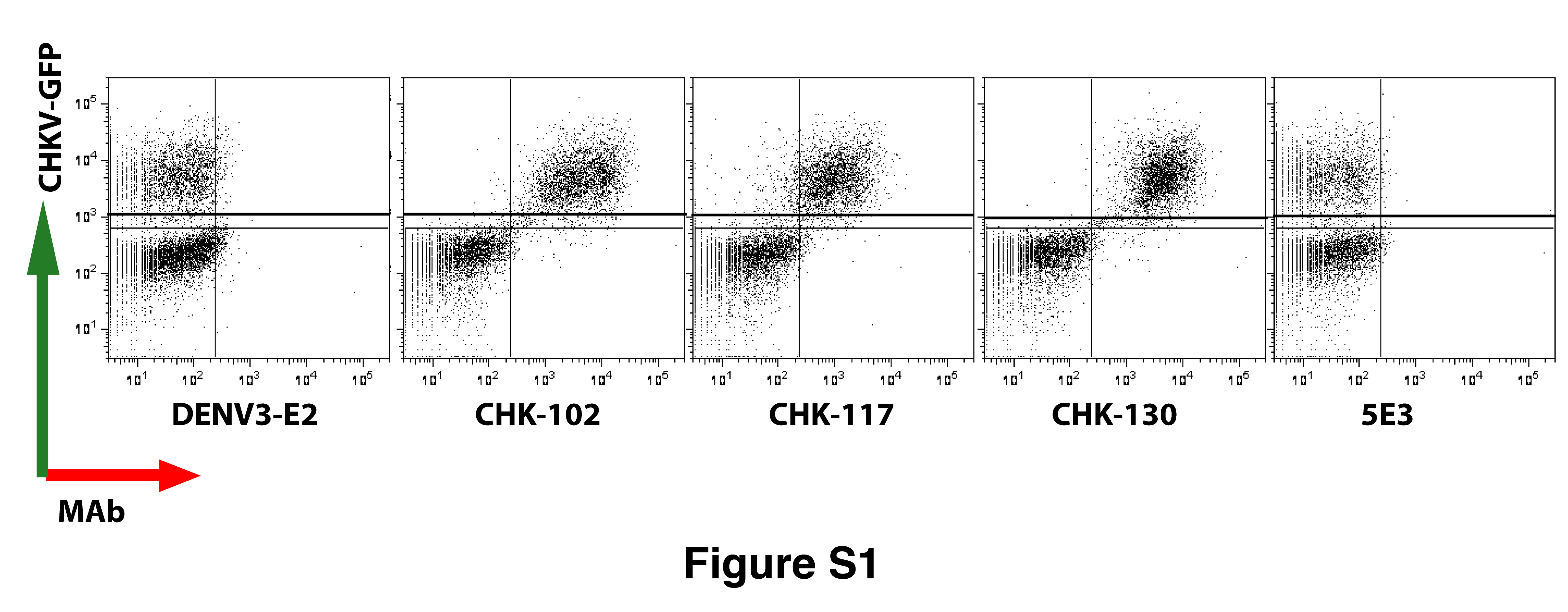

Supplement: Figure S1 — Screening of hybridoma supernatants for binding to CHIKV-infected cells. Hybridoma supernatants were incubated with CHIKV-GFP infected BHK21 cells and tested for immunoreactivity by flow cytometry. Shown are examples of a negative control MAb (DENV3-E2), three ‘hits’ (later named as CHK-102, CHK-117, and CHK-130), and a negative supernatant (5E3). The y-axis shows GFP staining associated with the reporter gene that is translated from the subgenomic promoter of CHIKV, and the x-axis shows staining of the tested mouse MAb. Double-positive cells were considered ‘hits’ in the screen. The result is representative of many different MAbs performed in the original screen. (TIF) [file ppat.1003312.s001.tif]

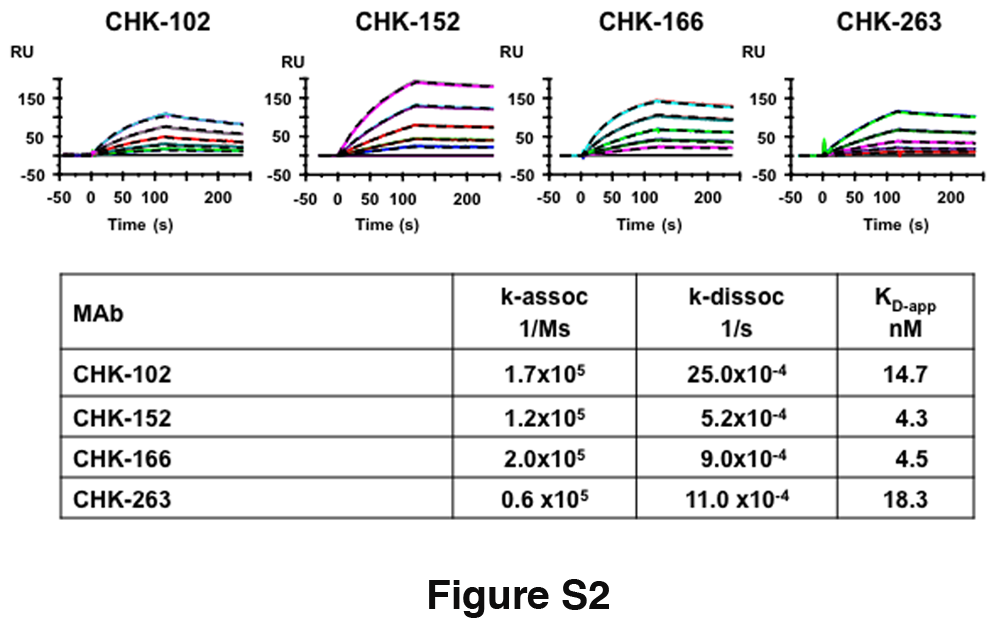

Supplement: Figure S2 — Binding kinetics of CHK-MAbs to pE2-E1. Binding curves and kinetic parameters of pE2-E1 binding to mouse CHK-102, CHK-152, CHK-166, and CHK-263 MAbs. A single representative sensogram is shown for each MAb. The experimental curves (colored lines) were fit using a 1∶1 Langmuir analysis (dashed lines), after double referencing, to determine the kinetic parameters presented immediately below. (TIF) [file ppat.1003312.s002.tif]

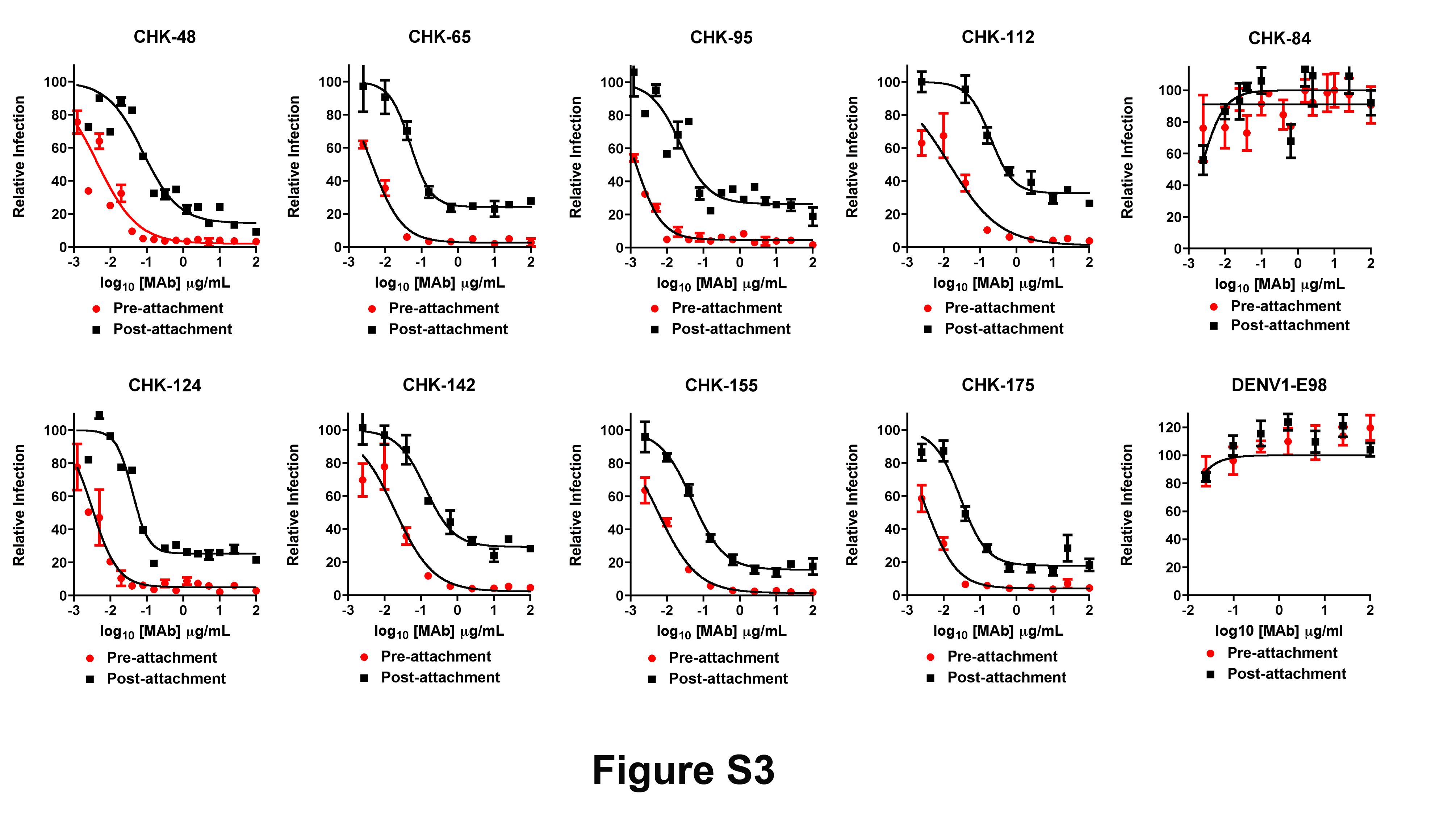

Supplement: Figure S3 — Pre- and post-attachment neutralization assays. Vero cells were pre-chilled to 4°C and 100 FFU of CHIKV-LR was added to each well for one hour at 4°C. After extensive washing at 4°C, the indicated MAbs (CHK-48, CHK-65, CHK-95, CHK-112, CHK-124, CHK-142, CHK-155, CHK-175, CHK-84 and DENV1-E98) were added for one hour at 4°C, and then the FRNT protocol was completed (black lines, Post). In comparison, a standard pre-incubation FRNT with all steps performed at 4°C is shown for reference. Virus and MAb are incubated together for one hour at 4°C, prior to addition to cells (red lines, Pre). Data shown are representative of three experiments performed in duplicate with error bars representing standard deviation. (TIF) [file ppat.1003312.s003.tif]

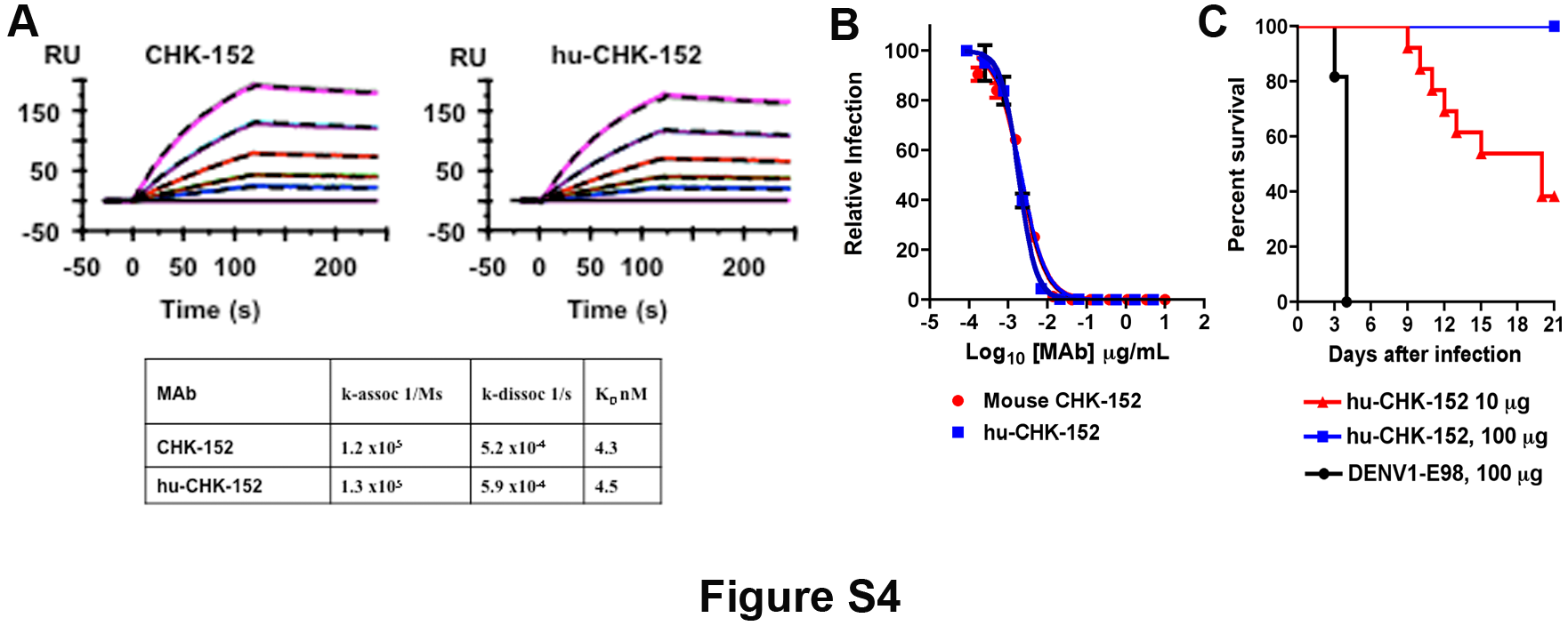

Supplement: Figure S4 — Construction and efficacy of humanized CHK-152. We amplified the cDNA encoding the heavy (VH) and light (VL) variable domains from the hybridoma cellular RNA and grafted the complementarity determining regions onto the human VH1-18 and human Vκ-L6 backbones. The resulting humanized VH and VL were combined with human γ1 and κ constant regions, fused to an IgG signal sequence, expressed in 293T cells and purified (data not shown). A. Binding curves and kinetic parameters of pE2-E1 binding to mouse CHK-152 and hu-CHK-152. A single representative sensogram is shown for each MAb. The experimental curves (colored lines) were fit using a 1∶1 Langmuir analysis (dashed lines), after double referencing, to determine the kinetic parameters presented in the Table immediately below. B. Neutralization studies with mouse CHK-152 and hu-CHK-152. Neutralizing activity was determined by FRNT assay on Vero cells. Samples were performed in duplicate and the experiment is one representative of three. C. Pre-exposure protective activity of hu-CHK-152. Ifnar −/− mice were passively transferred via an i.p. injection 10 or 100 µg of mouse hu-CHK-152 one day before CHIKV infection. Mice were monitored for survival for 21 days after infection. The survival curves were constructed from data of at least two independent experiments and the number of animals for each antibody ranged from 8 to 10 per group. (TIF) [file ppat.1003312.s004.tif]

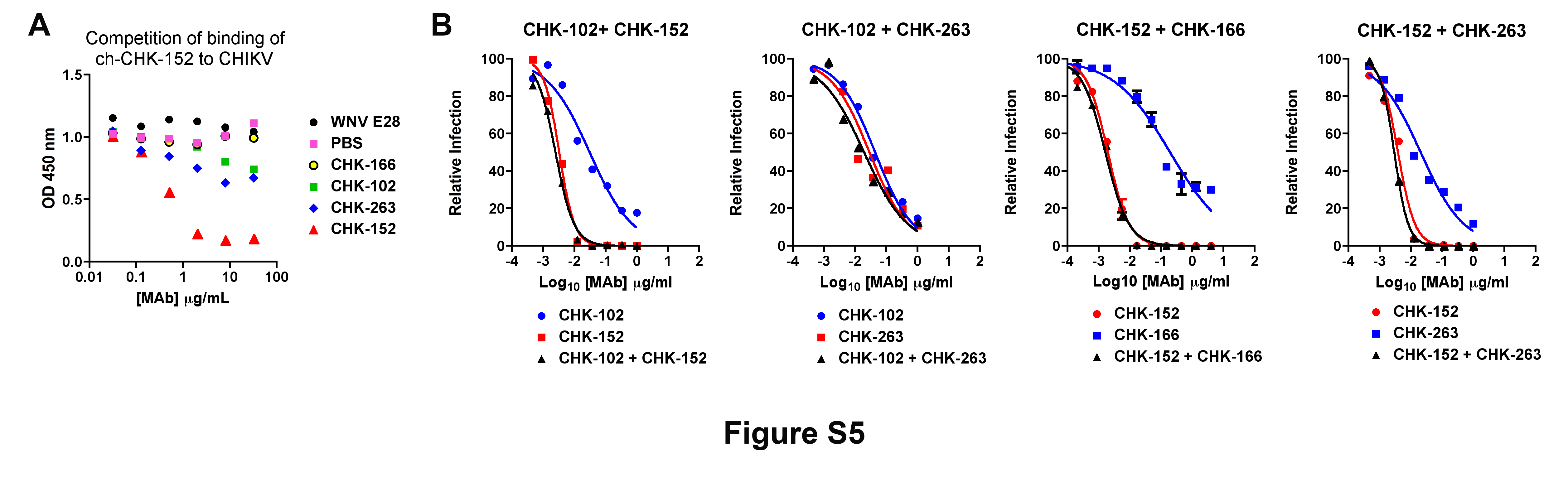

Supplement: Figure S5 — Interaction of neutralizing MAbs. A. Virion capture ELISA and competition of MAb binding. 96-well plates were coated with 5 µg/ml of CHK-65 MAb. Non-specific binding sites were blocked, and 3×10^6^ FFU of CHIKV 181-25 was captured. Subsequently, plates were incubated with the indicated anti-CHK mouse MAbs (CHK-102, CHK-152, CHK-166, or CHK-263) or controls (no MAb, PBS; irrelevant MAb, WNV E28) for one hour. After washing, plates were incubated sequentially with 125 ng/ml hu-CHK-152 and biotin-labeled goat anti-human secondary antibody. After washing and incubation with HRP-conjugated streptavidin, plates were developed and emission (450 nm) was read using an iMark microplate reader (Bio-Rad). Results are representative of three independent experiments, each performed in triplicate. B. Neutralizing activity of MAb combinations. Increasing concentrations of individual MAbs (CHK-102, CHK-152, CHK-166, and CHK-263) or combinations of MAbs (CHK-102+CHK-152, CHK 102+CHK-263, CHK-152+CHK-166, or CHK-152+CHK-263) were mixed with 100 FFU of CHIKV-LR for one 1 hour at 37°C and Vero cells were infected. Neutralization was determined by FFU assay. Data is representative of three independent experiments performed in duplicate. (TIF) [file ppat.1003312.s005.tif]

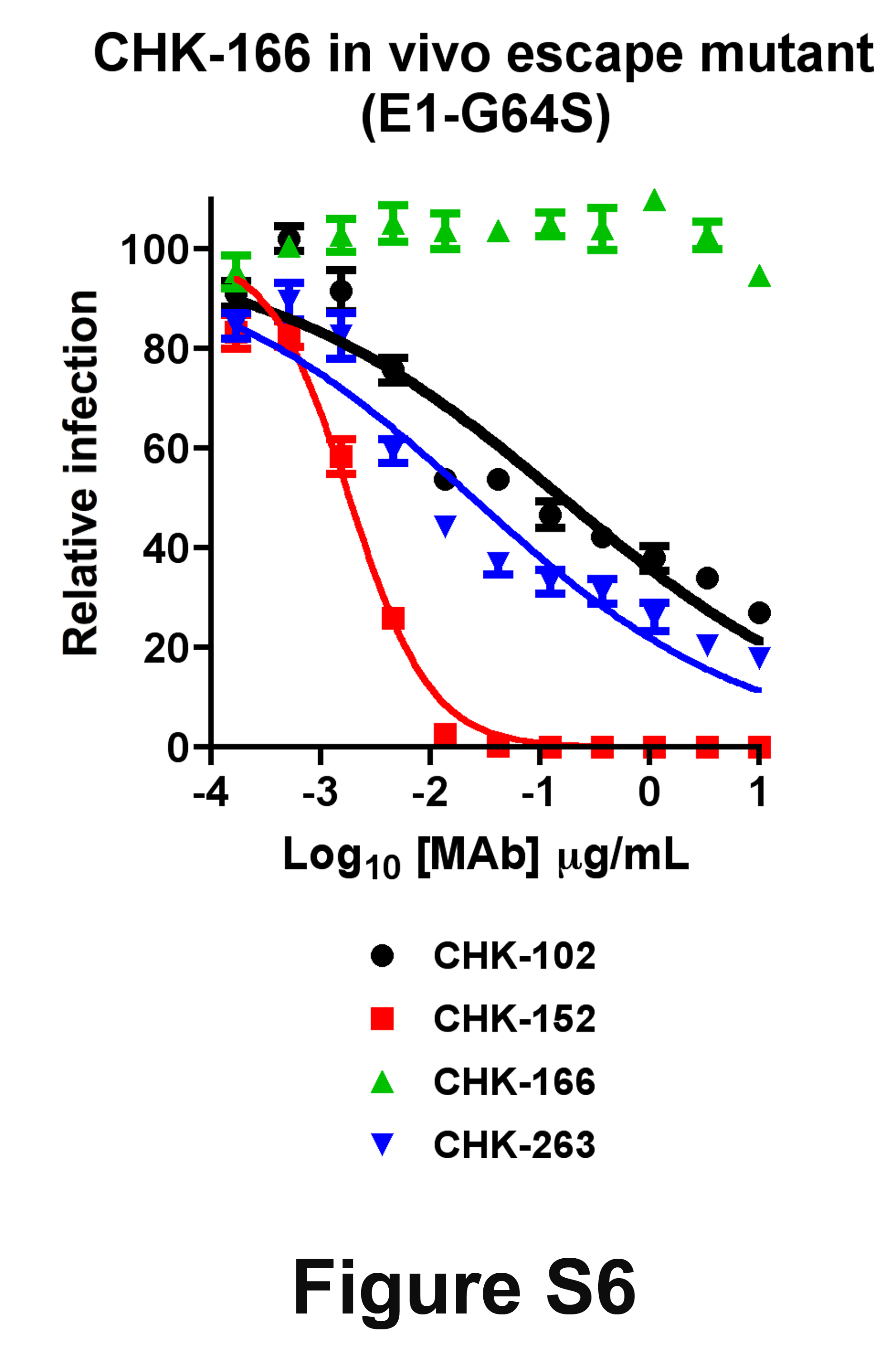

Supplement: Figure S6 — Selection of escape E1-G64S escape mutant in vivo against CHK-166. Ifnar −/− mice were infected with CHIKV and 24 hours later administered a single 100 µg dose of CHK-166. Six days later, virus was recovered from the contralateral leg and brain from one moribund mouse and the structural genes were sequenced. Both viral isolates recovered showed a single point G64S mutation in the E1 gene. This isolate was tested for neutralization by CHK-102 (EC50 of 161 ng/ml), CHK-152 (EC50 of 2 ng/ml), CHK-166 (EC50>10,000 ng/ml) and CHK-263 (25 ng/ml). Data is the average of two independent experiments performed in triplicate. (TIF) [file ppat.1003312.s006.tif]

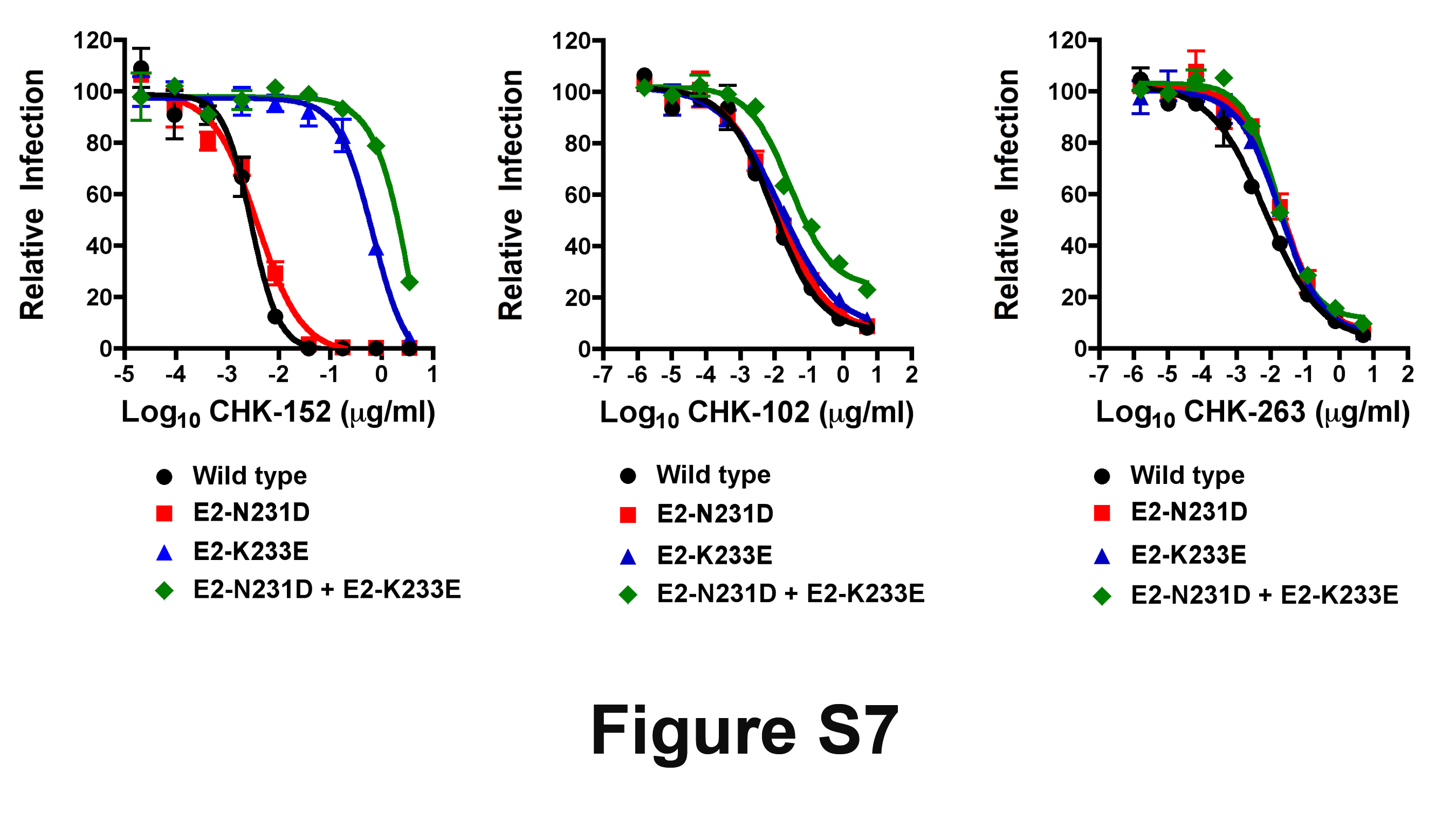

Supplement: Figure S7 — Confirmation of neutralization escape mutants selected in vivo . Confirmation of resistant phenotype selected with CHK-152 in vivo using SFV-CHIKV-GFP containing the indicated single engineered point mutations. Serial dilutions of CHK-152, CHK-102, and CHK-263 were incubated with chimeric SFV-CHIKV virus (WT or mutant stocks) for one hour at room temperature. MAb-virus complexes were added to Vero cells plated in 96-well plates and incubated at 37°C. After 8 hours cells were trypsinized, fixed, and the number of GFP-positive infected cells was assessed by flow cytometry. Curves are representative of 2 independent experiments. (TIF) [file ppat.1003312.s007.tif]

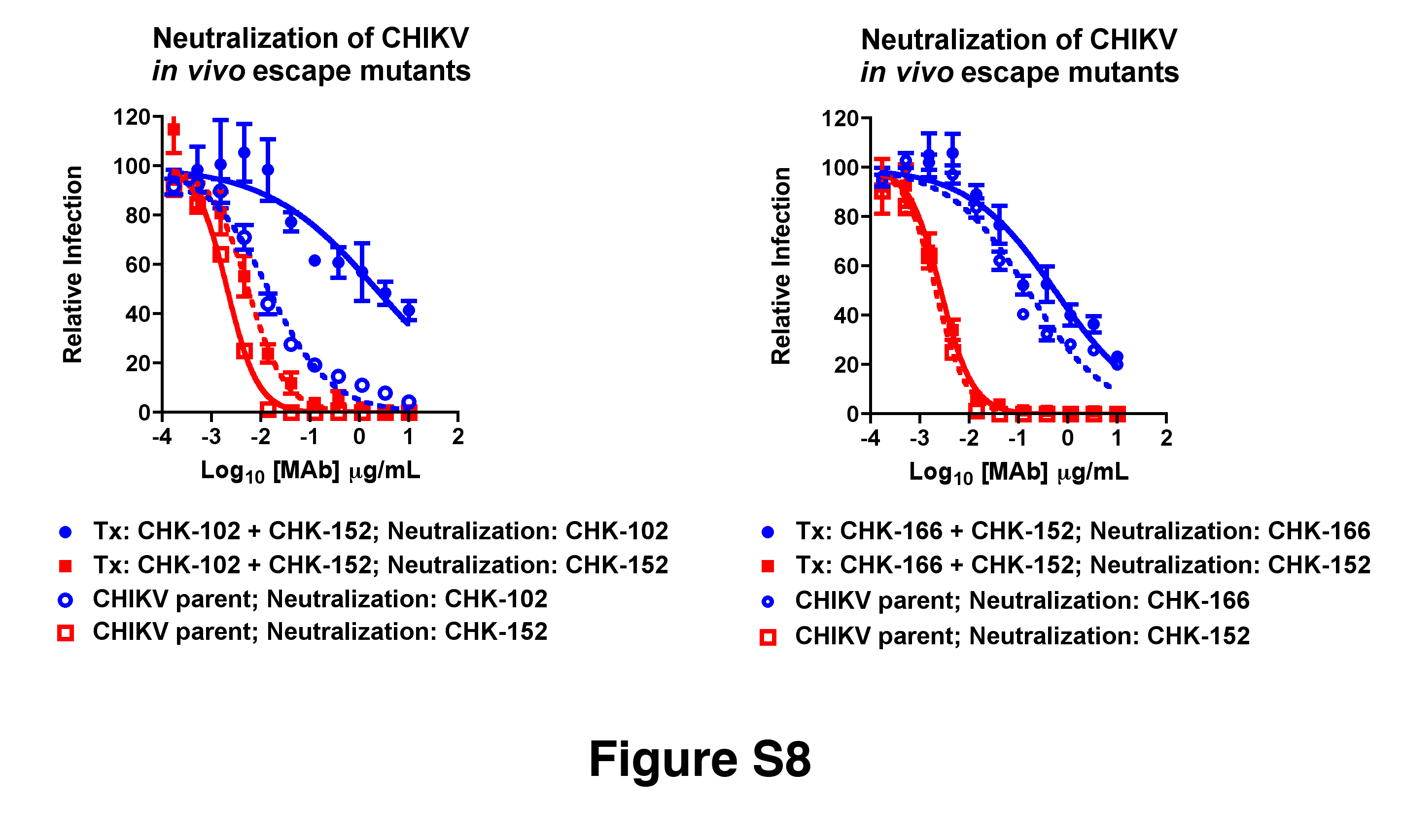

Supplement: Figure S8 — Relative resistance of CHIKV recovered from mice after treatment with combination MAb therapy. Ifnar −/− mice were infected with CHIKV and 48 hours later given a single dose of combination MAb (CHK-102+CHK-152 or CHK-166+CHK-152) therapy. Virus was recovered from the contralateral leg and/or brain from the few moribund mice and the structural genes were sequenced. Two viral isolates showed differences in neutralization patterns that corresponded to amino acid substitutions (see Table 2 ). Neutralization analysis of these viruses recovered from animals treated with (left) CHK-102 and CHK-152 or (right) CHK-166 and CHK-152 and tested against the respective MAbs. A comparison with the parent virus is shown. The curves are representative of two independent experiments performed in triplicate, and error bars indicate standard deviations. (TIF) [file ppat.1003312.s008.tif]
