# Supplementary material for: Development of a Highly Protective Combination Monoclonal Antibody Therapy against Chikungunya Virus
Source: PLoS Pathog. 2013 Apr 18;9(4):e1003312. doi: 10.1371/journal.ppat.1003312 (PMC3630103; doi:10.1371/journal.ppat.1003312)
Supplement: Text S1 — Supplemental Methods and Tables S1–S4. (DOCX) [file ppat.1003312.s009.docx]

**SUPPORTING INFORMATION**

**SUPPLEMENTAL METHODS**

**Humanization of CHK-152**. To construct a humanized version of CHK-152, a cDNA encoding the CDR from the V_H_ and V_L_ variable domains were amplified from hybridoma cell RNA by a 5' RACE procedure and grafted onto the homologous human V_H_ (1-18) and Vκ (L-6) backbones. The resulting humanized V_H_ and V_L_ were combined with human γ1 and κ constant regions, fused to an IgG signal sequence and inserted into a pCI-neo cassette to construct the heavy and light chain expression plasmids.

**Competition ELISA** Polystyrene 96-well plates were coated overnight at 4°C with 5 µg/ml of CHK-65 MAb in sodium carbonate buffer (pH 9.3). Plates were washed three times in PBS with 0.02% Tween 20 and blocked for one hour at 37°C with PBS, 2% BSA, and 0.02% Tween 20. 3 X 10^6^ FFU of CHIKV 181-25 (gift of R. Tesh, Galveston, TX) was captured, and plates were washed four times with PBS with 1% BSA and incubated with murine MAbs for one hour. After washing, plates were incubated with 125 ng/ml hu-CHK-152 in PBS with 1% BSA. Plates were washed and biotin-labeled goat anti-human secondary antibody (Jackson Labs) was added for one hour. After washing and incubation with HRP-conjugated streptavidin (Vector Laboratories), plates were developed with tetramethylbenzidine substrate (Dako). The reaction was stopped with the addition of 2 N H_2_SO_4_, and emission (450 nm) was read using an iMark microplate reader (Bio-Rad).

**Table S1. List of anti-CHIKV MAbs.**

| **MAb^a^** | **Isotype** | **E2^b^** | **MAb** | **Isotype** | **E2** | **MAb** | **Isotype** | **E2** | **MAb** | **Isotype** | **E2** |
| --- | --- | --- | --- | --- | --- | --- | --- | --- | --- | --- | --- |
| **CHK-1** | IgG2c | No | **CHK-59** | IgG2c | Yes | **CHK-127** | IgG1 | Yes | **CHK-206** | IgG1 | No |
| **CHK-2** | IgG2c | No | **CHK-60** | IgG2c | Yes | **CHK-129** | IgG2c | Yes | **CHK-207** | N.D. | N.D. |
| **CHK-3** | IgG2c | weak | **CHK-61** | IgG2c | Yes | **CHK-130** | IgG2b | Yes | **CHK-208** | IgM | No |
| **CHK-4** | IgG2c | Yes | **CHK-62** | IgG2c | Yes | **CHK-131** | IgG2c | Yes | **CHK-209** | N.D. | N.D. |
| **CHK-5** | IgG2b | Yes | **CHK-63** | IgG2b | Yes | **CHK-133** | IgG2b/c | Yes | **CHK-210** | N.D. | N.D. |
| **CHK-6** | IgG2c | Yes | **CHK-64** | IgG2c | Yes | **CHK-134** | IgG1 | Yes | **CHK-211** | N.D. | N.D. |
| **CHK-7** | IgG3 | weak | **CHK-65** | IgG2c | Yes | **CHK-135** | IgG2c | Yes | **CHK-212** | N.D. | N.D. |
| **CHK-8** | IgG2c | No | **CHK-66** | IgG2c | Yes | **CHK-138** | IgG2c | Yes | **CHK-213** | IgG2c | No |
| **CHK-9** | IgG2c | Yes | **CHK-67** | IgG2c | Yes | **CHK-140** | IgG2c | Yes | **CHK-214** | N.D. | N.D. |
| **CHK-10** | IgG2c | No | **CHK-68** | IgG2c | Yes | **CHK-141** | IgG2c | Yes | **CHK-215** | IgM/IgG2b | No |
| **CHK-11** | IgG2c | Yes | **CHK-69.A** | IgG1 | Yes | **CHK-142** | IgG2c | Yes | **CHK-216** | N.D. | N.D. |
| **CHK-12** | IgG2c | No | **CHK-69.B** | IgG2c | Yes | **CHK-143** | IgG3 | No | **CHK-217** | IgM | No |
| **CHK-13** | IgG1 | Yes | **CHK-70** | IgG2c | Yes | **CHK-144** | IgG2c | Yes | **CHK-218** | N.D. | Yes |
| **CHK-14** | IgG2c | Yes | **CHK-71** | IgG2c | Yes | **CHK-145** | IgG2c | Yes | **CHK-219** | N.D. | N.D. |
| **CHK-15** | IgM | No | **CHK-72** | IgG2c | Yes | **CHK-147** | IgG2b | Yes | **CHK-220** | N.D. | N.D. |
| **CHK-16** | N.D. | N.D | **CHK-73** | IgG2c | Yes | **CHK-148** | IgG2c | Yes. | **CHK-222** | IgG1 | No |
| **CHK-17** | IgG2c | Yes | **CHK-74** | IgG2c | Yes | **CHK-149** | IgG2c | Yes | **CHK-223** | IgM | No |
| **CHK-18** | IgG2c | No | **CHK-75** | IgG1 | Yes | **CHK-150** | IgG2b | Yes | **CHK-224** | N.D. | N.D. |
| **CHK-19** | IgG2c | No | **CHK-76** | IgG1 | Yes | **CHK-151** | IgG2c | Yes | **CHK-225** | IgM | No |
| **CHK-20** | N.D. | N.D | **CHK-77** | IgG2c | Yes | **CHK-152** | IgG2c | No | **CHK-226** | IgM | No |
| **CHK-21** | IgM | No | **CHK-78** | IgG2b | Yes | **CHK-153** | N.D. | N.D. | **CHK-227** | N.D. | N.D. |
| **CHK-22** | IgG2b | No | **CHK-79** | IgM/IgG1 | Yes | **CHK-154** | N.D. | N.D. | **CHK-228** | N.D. | N.D. |
| **CHK-23** | N.D | N.D | **CHK-81** | IgG2c | Yes | **CHK-155** | IgG2c | Yes | **CHK-229** | IgG1/IgM | No |
| **CHK-24** | IgG1/IgM | No | **CHK-82** | IgG2c | Yes | **CHK-156** | IgG2b | Yes | **CHK-230** | IgG1 | No |
| **CHK-25** | IgG2c | Yes | **CHK-83** | IgG2c | Yes | **CHK-157** | IgG2b | Yes | **CHK-231** | IgG2c | No |
| **CHK-26** | N.D | N.D | **CHK-84** | IgG2c | Yes | **CHK-158** | IgG2c | Yes | **CHK-232** | IgM | No |
| **CHK-27** | N.D | No | **CHK-85** | IgG2c | Yes | **CHK-159** | IgG2c | Yes | **CHK-233** | IgM | No |
| **CHK-28** | N.D | No | **CHK-86** | IgG2c | Yes | **CHK-160** | IgG2c | Yes | **CHK-234** | IgG1 | No |
| **CHK-29** | N.D | No | **CHK-87** | IgG1 | Yes | **CHK-161** | IgG2c | Yes | **CHK-235** | IgG1 | No |
| **CHK-30** | IgM | No | **CHK-88** | IgG2c | Yes | **CHK-162** | IgG2c | Yes | **CHK-237** | IgM | No |
| **CHK-31** | N.D | No | **CHK-89** | IgG2b | Yes | **CHK-163** | IgG2c | Yes | **CHK-238** | IgM | No |
| **CHK-32** | N.D | No | **CHK-90** | IgM | No | **CHK-164** | IgG2c | Yes | **CHK-239** | IgM | weak |
| **CHK-33** | IgG2c | No | **CHK-91** | IgG2c | Yes | **CHK-165** | IgG2c | Yes | **CHK-240** | IgM | No |
| **CHK-34** | IgM | No | **CHK-92** | IgG2c | No | **CHK-166** | IgG2c | No | **CHK-241** | N.D. | N.D. |
| **CHK-35** | N.D | No | **CHK-94** | IgG2c | Yes | **CHK-168** | IgG2c | Yes | **CHK-242** | N.D. | N.D. |
| **CHK-36** | N.D | Yes | **CHK-95** | IgG2c | Yes | **CHK-169** | IgG2c | Yes | **CHK-243** | N.D. | Yes |
| **CHK-37** | N.D | No | **CHK-96** | IgG2c | Yes | **CHK-170** | IgG1 | No | **CHK-244** | IgG2c | Yes |
| **CHK-38** | IgG2c | No | **CHK-97** | IgG2c | Yes | **CHK-172** | IgG2c | Yes | **CHK-245** | N.D. | weak |
| **CHK-39** | N.D | N.D | **CHK-98** | IgG2c | Yes | **CHK-173** | IgG2c | Yes | **CHK-246** | IgG2c | Yes |
| **CHK-40** | IgG3 | No | **CHK-99** | IgG2c | Yes | **CHK-174** | IgG2b | Yes | **CHK-247** | IgG2c | Yes |
| **CHK-41** | N.D | N.D | **CHK-101** | IgG2b | Yes | **CHK-175** | IgG2c | Yes | **CHK-248** | N.D. | N.D. |
| **CHK-42** | N.D | N.D | **CHK-102** | IgG2c | Yes | **CHK-176** | IgG2c | No | **CHK-249** | N.D. | N.D. |
| **CHK-43** | N.D | N.D | **CHK-103** | IgM | No | **CHK-177** | IgG2c | Yes | **CHK-250** | N.D. | N.D. |
| **CHK-44** | N.D | N.D | **CHK-104** | IgG2c | Yes | **CHK-178** | IgG2c | Yes | **CHK-251** | IgG2c | Yes |
| **CHK-45** | N.D | N.D | **CHK-105** | IgG1 | Yes | **CHK-179** | IgG2c | Yes | **CHK-256** | IgM | No |
| **CHK-46** | IgG3 | No | **CHK-106** | IgG2c | Yes | **CHK-180** | IgG2c | Yes | **CHK-261** | N.D. | No |
| **CHK-47** | N.D | N.D | **CHK-107** | IgG2c | Yes | **CHK-181** | IgG1 | Yes | **CHK-262** | IgG2c | Yes |
| **CHK-48** | IgG2c | Yes | **CHK-108** | N.D. | No | **CHK-182** | N.D. | No | **CHK-263** | IgG2c | Yes |
| **CHK-49** | N.D | No | **CHK-109** | IgG1 | Yes | **CHK-186** | IgG2c | Yes | **CHK-264** | IgG2c | Yes |
| **CHK-50** | N.D | N.D | **CHK-110** | IgG2c | Yes | **CHK-187** | IgG2c | Yes | **CHK-265** | IgG2c | Yes |
| **CHK-51** | N.D | N.D | **CHK-112** | IgG2c | Yes | **CHK-188** | IgG2c | Yes | **CHK-266** | IgG2c/IgG1 | Yes |
| **CHK-52** | IgG1 | No | **CHK-114** | IgG2c | No | **CHK-189** | IgG2c | Yes | **CHK-267** | IgG2c | Yes |
| **CHK-53** | IgG2c | Yes | **CHK-117** | IgG2c | Yes | **CHK-190** | IgG2c | Yes | **CHK-268** | IgG2c | Yes |
| **CHK-54** | IgG2c | Yes | **CHK-119** | IgG2c | Yes | **CHK-191** | IgG2c | Yes | **CHK-269** | IgG2b | No |
| **CHK-55** | IgG2c | Yes | **CHK-122** | IgM | No | **CHK-193** | IgG2c | Yes | **CHK-270** | IgG2c | Yes |
| **CHK-56** | IgG2c | Yes | **CHK-124** | IgG2c | Yes | **CHK-201** | IgG1 | Yes |  |  |  |
| **CHK-57** | IgG2c | Yes | **CHK-125** | N.D. | No | **CHK-204** | IgG2b | Yes |  |  |  |
| **CHK-58** | IgG2c | Yes | **CHK-126** | N.D. | No | **CHK-205** | IgG2b | No |  |  |  |

All MAbs listed were cloned successfully by limiting dilution. Isotypes were assigned based on a commercial assay. MAbs CHK-1 to CHK-51 were produced from mice receiving a final boost with CHIKV VLP; MAbs CHK-52 to CHK-145 were produced from mice receiving a final boost with recombinant E2 protein; and MAbs CHK-146 to CHK-270 were produced from mice receiving a final boost with either recombinant E2 protein or infectious CHIKV-LR. Binding to soluble E2 or pE2-E1 expressed in bacteria or mammalian cells was determined by ELISA. N.D. indicates not determined. Yes, reflects binding that yielded an O.D. value of > 1.1; Weak, indicates binding with an O.D. value of > 0.8 and < 1.1; Background binding (irrelevant MAb control) had an O.D. value of 0.2.

**Table S2. Cross-neutralization of infection by wild type and mutant SFV-CHIKV infection with anti-CHIKV MAbs**

| **Selecting MAb** | **CHIKV variant** | **Test MAb** | **Test MAb** | **Test MAb** | **Test MAb** |
| --- | --- | --- | --- | --- | --- |
|  |  | **CHK-102** | **CHK-152** | **CHK-166** | **CHK-263** |
| CHK-102 | E2 L210P | **Resistant** | Sensitive | N.D. | **Resistant** |
| CHK-102 | E2 G209E | **Resistant** | Sensitive | N.D. | **Resistant** |
|  |  |  |  |  |  |
| CHK-152 | E2 D59N | Sensitive | **Resistant** | N.D. | Sensitive |
| CHK-152 | E2 A89E | Sensitive | Sensitive | N.D. | Sensitive |
|  |  |  |  |  |  |
| CHK-166 | E1 K61T | N.D. | N.D. | **Resistant** | N.D. |
|  |  |  |  |  |  |
| CHK-263 | E2 K215E | **Resistant** | Sensitive | N.D. | **Resistant** |

CHIKV escape variants were selected in the presence of the indicated neutralizing MAbs. After sequencing of escape variants, the indicated amino acid substitutions were engineered into an infectious SFV-GFP-CHIKV chimeric virus for analysis of resistance or sensitivity to neutralization. Viruses denoted as “resistant” were not neutralized appreciably by the indicated MAbs, whereas viruses marked “sensitive” showed no greater than a 2-fold difference in EC50 values compared to the parent virus. Results are from two to four independent dose-response experiments performed in duplicate with nine serial dilutions of each MAb.

**Table S3**. **V_H_ and V_L_ nucleotide and protein sequences of protective anti-CHIKV MAbs**

***A. CHK-102***

**CHK-102-V_H_**

atgggatggagctgtatcatgttcttcctcctgtcaggaactgcaggtgtccaatccCAGGTTCAGCTGCAGCAGTCTGGGGCTGAGCTGGTGAAGCCTGGGGCCTCAGTGAAGATTTCCTGCAAAACTTCTGGCTACGCATTCAGTAGTTTCTGGATGCACTGGGTGAAGCAGAGGCCTGGAAAGGGTCTTGAGTGGATTGGACAGATTTATCCTGGAGATGGTGATACTAACTATAACGGAAAGTTCAAGGACAAGGCCACACTGACTGCAGACAAATCCTCCAACACAGCCTACATGCAGCTCACCAGCCTGACCTCTGAGGACTCTGCGGTCTATTTCTGTGCAAGAAACTTACTTTTTGACTACTGGGGCCAAGGCACCACTCTCACAGTCTCCTCA

MGWSCIMFFLLSGTAGVQSQVQLQQSGAELVKPGASVKISCKTSGYAFSSFWMHWVKQRPGKGLEWIGQIYPGDGDTNYNGKFKDKATLTADKSSNTAYMQLTSLTSEDSAVYFCARNLLFDYWGQGTTLTVSS

**CHK-102-V_L_**

GAGATCCTGATGACTCAGTCTCCAGCCATCCTGTCTGTGAGTCCAGGAGAAAGAGTCAGTTTCTCCTGCAGGGCCAGTCAGAGCATTGGCTCAAACATACACTGGTATCAGCAAAGAACAAATGGTTCTCCAAGGCTTCTCATAAAGTATGCCTCTGAGTCTATCTCTGGGATCCCTTCCAGGTTTAGTGGCAGTGGGTCAGGGACAGATTTTACTCTTAGCATCAACAGTGTGGAGTCTGAAGATATTGCAGATTATTACTGTCAACAGAATAATATCTGGCCATTCACGTTCGGCTCGGGGACAAAGTTGGAAATAAAG

EILMTQSPAILSVSPGERVSFSCRASQSIGSNIHWYQQRTNGSPRLLIKYASESISGIPSRFSGSGSGTDFTLSINSVESEDIADYYCQQNNIWPFTFGSGTKLEIK

***B. CHK-152***

**CHK-152-V_H_**

atgggatggagctgtatcatcctcattttggtagcagcagctacaggtgtccactccCAGGTCCAGCTGCAGCAGCCTGGGGCTGCGCTTGTGAAGCCTGGGGCTTCAGCGATGATGTCCTGCAAGGCTTCTGGCTACACCTTCACCAGCTACTGGATAACCTGGGTGAAGCAGAGGCCTGGACAAGGCCTTGAATGGATTGGGGACATTTACCCTGGTACTGGTCGAACTATCTACAAGGAGAAGTTCAAGACCAAGGCCACACTGACTGTAGACACATCCTCCAGCACAGCCTTCATGCAGCTCAACAGCCTGACATCTGAGGATTCAGCGGTCTATTACTGTGCAAGAGGCTACGGTAGTCCTTACTATGCTTTGGACTACTGGGGTCAAGGAACCTCAGTCACCGTCTCCTCA

MGWSCIILILVAAATGVHSQVQLQQPGAALVKPGASAMMSCKASGYTFTSYWITWVKQRPGQGLEWIGDIYPGTGRTIYKEKFKTKATLTVDTSSSTAFMQLNSLTSEDSAVYYCARGYGSPYYALDYWGQGTSVTVSS

**CHK-152-V_L_**

atggagacagacacaatcctgctatgggtgctgctgctctgggttccaggctccactggtGACATTGTGCTGACCCAATCTCCAGCTTCTTTGGCTGTGTCTCAAGGGCAGAGGGCCACCATCTCCTGCaaggccagccaaagtgttgattatgatggtgatagttatgtgaacTGGTACCAACAGAAACCAGGACAGTCACCCAAACTCCTCATCTATgatgcatccaatctagaatctGGGATCCCAGCCAGGTTTAGTGGCAGTGGGTCTGGGACAGACTTCACCCTCAACATTCATCCTGTGGAGGAAGAGGATGTTGCAACCTATTACTGTcaggaaagtaatgaggatcctcggacgTTCGGTGGAGGCACCAAGCTGGAAATCAAA

METDTILLWVLLLWVPGSTGDIVLTQSPASLAVSQGQRATISCKASQSVDYDGDSYVNWYQQKPGQSPKLLIYDASNLESGIPARFSGSGSGTDFTLNIHPVEEEDVATYYCQESNEDPRTFGGGTKLEIK

***C. CHK-166***

**CHK-166-V_H_**

atgaacttggggctcagcttgattttccttgtccttgttttaaaaggtgtccagtgtGAAGTGAGGCTGGTGGAGTCTGGGGGAGGCTTAGAGCAGCCTGGAGGGTCCCTGAAACTCTCCTGTGCAGCCTCTGGATTCACTTTCAGTgactatttcatgtatTGGGTTCGCCAGACTCCAGAGAAGAGGCTGGAGTGGGTCGCAtatattagtaatggtggtattagtaccttttattcagacgctgttaagggcCGATTCACCATCTCCAGAGACAATGCCAGGAACACCCTATACCTACAAATGAGTCGTCTGAAGTCTGAGGACACAGCCATATATTACTGTGTAAGAcaggtctacggtcagggctactttgactacTGGGGCCAAGGCACCACTCTCGCAGTCTCCTCA

MNLGLSLIFLVLVLKGVQCEVRLVESGGGLEQPGGSLKLSCAASGFTFSDYFMYWVRQTPEKRLEWVAYISNGGISTFYSDAVKGRFTISRDNARNTLYLQMSRLKSEDTAIYYCVRQVYGQGYFDYWGQGTTLAVSS

**CHK-166-V_L_**

atggattttcaggtgcagattttcagcttcctgctaatcagtgcctcagtcataatgtccagaggaCAAATTGTTCTCATCCAGTCTCCAGCGATCATGTCTGCGTCTCTAGGGGAACGGGTCACCATGACCTGCACTGCCAGCTCAAGTGTAAGTTCCAGTTACTTGCACTGGTACCAGCAGAAGCCAGGATCCTCCCCCAAACTCTGGATTTATAGTTCATTCAGCCTGGCTTCTGGAGTCCCAGCCCGGTTCAGTGGCAGTGGATCTGGGACCTCTTACTCTCTCACAATCAGCACCATGGAGGCTGAAGATGCTGCCACGTATTACTGCCACCAGTATTTGCGTTCCCCGTGGACGTTCGGTGGAGGCTCCAAGCTGGAAATCAAA

MDFQVQIFSFLLISASVIMSRGQIVLIQSPAIMSASLGERVTMTCTASSSVSSSYLHWYQQKPGSSPKLWIYSSFSLASGVPARFSGSGSGTSYSLTISTMEAEDAATYYCHQYLRSPWTFGGGSKLEIK

***D. CHK-263***

**CHK-263-V_H_**

atggaatggcctttgatctttctcttcctcctgtcaggaactgcaggtgtccaatccCAGGTTCAGCTGCAGCAGTCTGGGGCTGAGCTGGTGAAGCCTGGGGCCTCAGTGAAGATTTCCTGCAAAGCTTCTGGCTACGCATTCAGTAGCTACTGGATGAACTGGGTGAAGCAGAGGCCTGGAAAGGGTCTTGAGTGGATTGGACAGATTTATCCTGGAGATGGTGATACTAACTACAACGGAAAGTTCAAGGGCAAGGCCACACTGACTGCAGACAAATCCTCCAGCACAGCCTACATGCAGCTCAGCAGCCTGACCTCTGAGGACTCTGCGGTCTATTTCTGTGCAAGAGGAGGTCTAACTATTGACTACTGGGGCCAAGGCACCACTCTCACAGTCTCCTCA

MEWPLIFLFLLSGTAGVQSQVQLQQSGAELVKPGASVKISCKASGYAFSSYWMNWVKQRPGKGLEWIGQIYPGDGDTNYNGKFKGKATLTADKSSSTAYMQLSSLTSEDSAVYFCARGGLTIDYWGQGTTLTVSS

**CHK-263-V_L_**

atggtatccacacctcagttccttggacttatgcttttttggatttcagcctccagaggtGATATTGTACTGACTCAGTCTCCAGCCACCCTGTCTGTGACTCCAGGAGATAGCGTCAGTCTTTCCTGCAGGGCCAGCCAAAGTATTAGCGACAACCTACACTGGTATCAACAAAAATCACATGAGTCTCCAGGGCTTCTCATCAAGTATGCTTCCCAGTCCATCTCTGGGATCCCCTCCAGGTTCAGTGGCAGTGGATCAGGGACAGATTTCACTCTCAGTATCAACAGTGTGGAGACTGAAGATTTTGGAATGTATTTCTGTCAACAGAGTAACAGCTGGCCGTACACGTTCGGAGGGGGGACCAAGCTGGAAATAAAA

MVSTPQFLGLMLFWISASRGDIVLTQSPATLSVTPGDSVSLSCRASQSISDNLHWYQQKSHESPGLLIKYASQSISGIPSRFSGSGSGTDFTLSINSVETEDFGMYFCQQSNSWPYTFGGGTKLEIK

The V_H_ and V_L_ variable domains were amplified from hybridoma cell RNA by a 5' RACE procedure. Underlined nucleotides and amino acids correspond to the signal sequences, when obtained.

**Table S4. Primers used for sequencing and amplifying the structural genes of CHIKV-LR 2006-OPY1.**

| **Sequencing Primer** | **5**'**-Sequence-3**' |
| --- | --- |
| 1: 5'-8248F | GTCTTAGGAGGAGCTAATGAAGGAG |
| 2: 5’-8573F | CCACAAGACCATACTTAGCTCACTGTCC |
| 3: 5'-8912F | CATGTACGCACCCATTTCACC |
| 4: 5'-9224F | CGGTCACCAATCACAAAAAGT |
| 5: 5'-9500F | CCGTGCCGACTGAAGGG |
| 6 5'-9802F | GCTAAAGCGGCCACATACC |
| 7: 5'-10101 | CACTTTGGAGCCAACACTATCG |
| 8: 5'-10389 | GCTCCGCGTCCTTTACCA |
| 9: 5'-10676 | CGGTACACGTGCCATACTCTCAGG |
| 10: 5'-11017 | GCTGAGATAGAAGTTGAAGGGA |

| **PCR Primer** | **5**'**-Sequence-3**' |
| --- | --- |
| 5'-8248F | GTCTTAGGAGGAGCTAATGAAGGAGCCCGT |
| 5'-11359R | GTGTGTCTCTTAGGGGACACATATACCTTCATACTT |
